# Emerging Zoonotic Sporotrichosis in Fortaleza, Brazil: Spatiotemporal Distribution, Exploratory Machine Learning Risk Modeling, and One Health Implications of *Sporothrix brasiliensis* Detection

**DOI:** 10.3390/ani16213498

**Published:** 2026-11-05

**Authors:** Barbara Wilka Leal Silva, Carlos Gabriel Oliveira Freitas, Davi Emanuel Ribeiro de Sousa, Juliana Paula Martins Alves, Camila de Oliveira Campos, Ana Raquel Almeida Pinheiro, Francisco Bastos Cavalcante Sobrinho, Adriana de Queiroz Pinheiro, Steffi Lima Araujo, Yury Carantino Costa Andrade, Francisco Antônio Félix Xavier Júnior, Isaac Neto Goes da Silva

**Affiliations:** 1 Laboratory of Medical Sciences (LACIMED), Faculty of Veterinary, State University of Ceará, Fortaleza 60714-903, CE, Brazil; raquel.pinheiro@aluno.uece.br (A.R.A.P.); bastos.sobrinho@aluno.uece.br (F.B.C.S.); steffi.lima@uece.br (S.L.A.); yurycarantinocosta@gmail.com (Y.C.C.A.); isaac.neto@uece.br (I.N.G.d.S.); 2 Laboratory of Intelligence Artificial and Logic, Federal University of Ceará, Fortaleza 60455-760, CE, Brazil; cgofreitas@alu.ufc.br; 3 School of Veterinary Medicine, Christus University, Fortaleza 60125-058, CE, Brazil; daviers@hotmail.com (D.E.R.d.S.); juli.alves@uece.br (J.P.M.A.); 4 Zoonosis Surveillance Unit, Fortaleza 60714-612, CE, Brazil; milaoc@gmail.com; 5 Laboratory of Microbiology and Analysis, Faculty of Veterinary, State University of Ceará, Fortaleza 60714-903, CE, Brazil; adriana.queiroz@uece.br; 6 Laboratory of Veterinary Clinical Pathology, Faculty of Veterinary, State University of Ceará, Fortaleza 60714-903, CE, Brazil

**Keywords:** *Sporothrix brasiliensis*, feline sporotrichosis, zoonoses, geographic analysis, machine learning, One Health, public health

## Abstract

Zoonotic sporotrichosis caused by *Sporothrix brasiliensis* is an emerging public health threat in Brazil. Under a One Health perspective, this study described the temporal and geographic distribution of recorded feline sporotrichosis detections in Fortaleza (2022–2026), as well as their co-occurrence with human notifications. Additionally, the study explored associations with socio-environmental factors and evaluated predictive performance at both the neighborhood and individual-animal levels. Suspected cats underwent cytology, culture, and species-specific calmodulin PCR. Cases were mapped across 116 neighborhoods, and logistic regression and random forest models were evaluated exploratorily at the neighborhood (61 neighborhoods) and animal (239 cats) levels, using grouped nested cross-validation and Spearman correlations corrected for multiple testing. Of 246 cats, 120 were confirmed, and all 37 isolates analyzed were *S. brasiliensis*. Neighborhoods with cases rose from one (2022) to 34 (March 2026), with 68 human notifications in 25 neighborhoods. After grouped validation, none of the evaluated predictor–outcome contrasts met the significance criterion after BY adjustment, and the exploratory associations were subject to substantial uncertainty. Consequently, no model demonstrated meaningful discriminative performance (out-of-sample AUC-ROC 0.34–0.50 at the neighborhood level and 0.38–0.47 at the animal level). These results are consistent with the complex, multifactorial nature of the disease, in which host behavior, environmental persistence, and surveillance intensity likely interact. These findings confirm the urban establishment of *S. brasiliensis* and the need for more complete data and robust analytical approaches. Outbreak control requires integrated One Health strategies: expanded access to diagnosis, cat neutering, and strengthened surveillance.

## Simple Summary

Feline sporotrichosis, caused by *Sporothrix brasiliensis*, is an emerging zoonotic threat in South America. We investigated the spread of the disease among cats in Fortaleza, Brazil, between January 2022 and March 2026. Among 246 suspected cats, 120 were confirmed positive, with cases expanding from 1 to 34 neighborhoods. Machine learning models failed to accurately predict disease occurrence better than chance, reflecting the complex, multifactorial nature of sporotrichosis—where host behavior, environmental persistence, and variable surveillance intensity interact. These findings demonstrate the active urban establishment of the fungus and emphasize the need for more robust analytical approaches. Controlling this outbreak requires integrated One Health strategies, including expanded diagnostic access, widespread cat neutering, and improved surveillance infrastructure.

## 1. Introduction

Sporotrichosis is a subcutaneous mycosis caused by dimorphic fungi of the genus *Sporothrix*, recognized as one of the most clinically and veterinary significant implantation mycoses globally [1]. Within the *Sporothrix schenckii* complex, *Sporothrix brasiliensis* stands out due to its high virulence, remarkable adaptation to feline hosts, and propensity for zoonotic transmission [2,3]. The exceptionally high fungal burden found in the cutaneous and mucosal lesions of infected cats facilitates the dissemination of the agent to humans and other animals via scratches, bites, or direct contact with contaminated exudates [1,2,3,4].

Over the past two decades, Brazil has consolidated its position as the global epicenter of zoonotic sporotrichosis driven by *S. brasiliensis* [1]. Initially restricted to the southeastern region, particularly the state of Rio de Janeiro, the disease has undergone aggressive geographic expansion, leading to a dramatic surge in both animal epizootics and human cases across several states [3,5].

Recent epidemiological and molecular investigations have confirmed the consolidation of *S. brasiliensis* in the Brazilian Northeast, reaching states such as Rio Grande do Norte, Paraíba, Pernambuco, and Bahia [6,7]. In Pernambuco, for instance, more than 1100 feline cases were registered over a five-year period; molecular typing revealed extremely low genetic diversity and high clonal similarity with strains originating from Rio de Janeiro, pointing to a recent, rapid clonal expansion and dispersal in the region [8]. Similar dynamics of territorial expansion and new epidemic focus establishment have been observed in neighboring northeastern states [9,10,11]. In the state of Ceará, the first reports of both human and feline sporotrichosis were described only recently in its capital, Fortaleza, marking the establishment of the disease in a territory historically considered non-endemic [12,13].

Fortaleza presents a highly complex socio-ecological ecosystem for zoonotic transmission. As one of the most densely populated metropolises in Northeastern Brazil, the city is characterized by intense urbanization coupled with stark socio-environmental inequalities, high stray and semi-domesticated feline densities, and precarious sanitary infrastructure in its peripheral neighborhoods [14,15,16].

In this context, spatial description and predictive modeling provide complementary perspectives on the epidemiology of feline sporotrichosis [17,18]. While geographic mapping supports the visualization of territorial patterns, machine learning algorithms, such as Binary Logistic Regression and Random Forest, offer robust frameworks to examine how specific socio-environmental and individual-level predictors contribute to disease occurrence [19,20,21,22]. Applying these algorithms independently at each level (neighborhood and individual animal) avoids combining, within a single model, variables measured at different ecological scales [23,24].

Despite the recent recognition of zoonotic sporotrichosis in Fortaleza, the spatio-temporal dynamics of feline cases and their relationship with local socio-environmental vulnerabilities remain insufficiently characterized. Therefore, this study aimed to describe the temporal and territorial distribution of culture-confirmed feline sporotrichosis cases in Fortaleza between January 2022 and March 2026 alongside their geographic co-occurrence with human cases. Additionally, we identified the species among molecularly tested isolates and evaluated the predictive performance of Binary Logistic Regression and Random Forest models. By conducting independent analyses at both the neighborhood and individual-animal levels, we aim to provide evidence-based insights for targeted surveillance and control strategies under a One Health perspective.

## 2. Materials and Methods

### 2.1. Ethical Statement, Study Area and Data Sources

This study was conducted in strict accordance with the Brazilian National Health Council and approved by the Institutional Ethics Committee on Animal Use (CEUA) under protocol number 31032.004491/2025-81.

The study was conducted in the municipality of Fortaleza, the capital of the state of Ceará, Northeastern Brazil (3°43′ *S*; 38°32′ *W*). Fortaleza encompasses a territorial area of approximately 312 km^2^ and has an estimated human population exceeding 2.4 million inhabitants, representing one of the most densely populated urban centers in the country [14]. The municipality is administratively divided into 121 neighborhoods [25] and 12 regional executive secretariats; however, 116 neighborhoods were considered in this study owing to data collection constraints and geographical proximity (Table S1). All neighborhoods included in the study are located within the municipality of Fortaleza, which has a hot, sub-humid tropical climate [15]. This municipal climatic characterization is provided as environmental context for the study area. Microclimatic differences among neighborhoods were not assessed.

A retrospective observational study was conducted to evaluate the spatiotemporal distribution of feline sporotrichosis cases recorded between January 2022 and March 2026. Epidemiological data on feline cases were obtained from the Zoonosis Surveillance Unit (Unidade de Vigilância de Zoonoses—UVZ) of Fortaleza, the municipal reference center for sporotrichosis surveillance. Suspected feline cases and their diagnostic samples reached the UVZ through both passive surveillance and active case finding. Passive surveillance comprised the spontaneous presentation of suspected cats by their owners and the referral of animals initially attended at public animal health services or private veterinary clinics. Active case finding was conducted by endemic disease control agents through house-to-house visits performed as part of municipal strategies already established for the surveillance, control, and notification of other zoonoses. Cats identified during these visits as having clinical or epidemiological suspicion of sporotrichosis were referred for diagnostic investigation.

Due to the progression in the number of positive diagnoses during the study period, the strategies for feline epidemiological surveillance underwent adaptations. Until the end of 2024, case detection was strictly reliant on passive surveillance, as the disease had only recently been introduced to the municipality. During 2025, an increase in spontaneous demand for veterinary care by pet owners in specific neighborhoods prompted the UVZ to initiate localized active case-finding interventions in those affected areas. Subsequently, starting in January 2026, active case-finding operations were systematically implemented across a broader area of the municipality.

Cats exhibiting clinical signs compatible with subcutaneous mycoses, including cutaneous ulcers, nodules, and respiratory signs [3], or contact with a culture-confirmed case that were identified through either surveillance route and submitted to diagnostic investigation were included in the study. Epidemiological data were collected using standardized questionnaires and included neighborhood of residence, sex, neutering status, kitten status (age ≤ 6 months), rearing system (strictly indoor or semi-domiciled/free roaming), contact with infected animals, and the presence of other animals with suspected sporotrichosis in the household. Animal hoarding was operationally defined as ≥5 animals per household. Cutaneous lesion distribution was classified as localized (≤2 lesions at the same anatomical site) or disseminated (>2 lesions at distinct anatomical sites). Animals without a description of the lesions, including one cat without visible lesions, were classified as “not reported”. Human sporotrichosis records from the corresponding study period were obtained from the Daily Disease Monitoring System (Sistema de Monitoramento Diário de Agravos—SIMDA) of the Municipal Health Secretariat of Fortaleza to assess the spatial and temporal overlap between human and feline cases [26]. Human case ascertainment relied predominantly on passive surveillance.

Case detection in both populations may have been limited by the recent emergence of sporotrichosis in the municipality and insufficient recognition of its characteristic lesions, while stigma surrounding affected cats may have further reduced feline case reporting. The dataset may therefore be affected by underreporting and ascertainment bias and should be interpreted as representing detected cases rather than the total disease burden or population-based incidence. Nevertheless, it reflects the surveillance framework currently available for a recently recognized municipal health concern without dedicated surveillance sectors or fully structured case-detection pathways.

### 2.2. Epidemiological Indicators

To characterize temporal patterns in surveillance-based case detection, the numbers of clinically suspected cats investigated (*S_y_*) and culture-confirmed feline cases (*C_y_*) were summarized for each study period. Data from 2022 through 2025 were summarized by year, whereas data for 2026 covered January through March only. Since cases were identified through passive referral and active case finding without a population-based sampling frame, population incidence and epidemic doubling time were not estimated [27].

The overall laboratory confirmation proportion for January 2022–March 2026 was calculated as:

$${\mathrm{LCP}}_{\mathrm{overall}}\;=\;\frac{C}{S}\;\times \;100$$

where *C* is the total number of culture-confirmed feline cases and *S* is the total number of cats investigated during the study period. The corresponding 95% confidence interval was estimated using the Wilson score method [28,29].

(1) $${\mathrm{CI}}_{95\%}\;=\;\frac{\hat{p}\;+\;\frac{z^{2}}{2n}\;\pm \;z\sqrt{\frac{\hat{p}\left(1\;-\;\hat{p}\right)}{n}\;+\;\frac{z^{2}}{4n^{2}}}}{1\;+\;\frac{z^{2}}{n}}$$

where $\hat{p}\;=\;C/S$, *n* = *S*, and *z* = 1.96.

The territorial coverage of recorded surveillance detections was assessed as the proportion of the municipality’s neighborhoods from which at least one clinically suspected cat or at least one culture-confirmed feline case was recorded:

(2) $${TC}_{\mathrm{suspected}}\;=\;\left(\frac{B_{S}}{B_{T}}\right)\;\times \;100$$

(3) $${TC}_{\mathrm{confirmed}}\;=\;\left(\frac{B_{C}}{B_{T}}\right)\;\times \;100$$

where *B_S_* is the number of neighborhoods with at least one clinically suspected cat, *B_C_* is the number of neighborhoods with at least one culture-confirmed feline case, and *B_T_* is the total number of standardized neighborhoods considered as territorial units in the municipality.

The spatial distribution of detected cases was described using neighborhood-specific case counts among records with valid neighborhood information. Records with missing neighborhood information were included in the overall and period-specific analyses but excluded from the spatial analysis.

The temporal progression of recorded geographic case detection was described using the number of neighborhoods in which a first laboratory-confirmed feline case was identified during each study period (*B*_new,_*_y_*). The cumulative number of neighborhoods with recorded case detection was calculated as:

(4) $$B_{\mathrm{cum},y}\;=\;\sum_{j=1}^{y}B_{\mathrm{new},j}$$

Cumulative territorial coverage was calculated as:

(5) $${\mathrm{CTC}}_{y}\;=\;\left(\frac{B_{\mathrm{cum},y}}{B_{T}}\right)\;\times \;100$$

where *B*_cum,_*_y_* is the cumulative number of neighborhoods with at least one laboratory-confirmed feline case by period *y*, and *B_T_* denotes the 116 standardized territorial units included in the study database after the mergers listed in Table S1, rather than the 121 official neighborhoods of the municipality. At the end of the study period, *TC*_confirmed_ corresponded to the final cumulative territorial coverage. These measures describe the temporal and geographic progression of recorded case detection within the surveillance system and should not be interpreted as estimates of population incidence, total disease burden, or epidemic growth.

The mean monthly number of neighborhoods with a first recorded culture-confirmed feline case was calculated as:

(6) $$D_{y}\;=\;\frac{B_{\mathrm{new},y}}{m_{y}}$$

where *B*_new,_*_y_* is the number of neighborhoods recording their first culture-confirmed feline case during period *y*, and *m_y_* is the number of months in that period: 12 for 2025 and three for January–March 2026.

### 2.3. Mycological and Molecular Identification

Exudates from cutaneous ulcers or mucosal swabs were collected for laboratory diagnosis. Cytological smears were prepared via imprint or scraping, stained using standard methods, and examined under light microscopy for yeast-like structures compatible with *Sporothrix* spp. For fungal isolation, samples were inoculated onto Sabouraud Dextrose Agar supplemented with chloramphenicol and cycloheximide and incubated at 25 °C for up to 35 days at the Medical Sciences Laboratory (LACIMED), State University of Ceará (UECE).

To assess thermal dimorphism, all filamentous isolates obtained at 25 °C (*n* = 120) were subcultured in brain heart infusion (BHI) medium and incubated at 37 °C for 7 days to induce conversion to the yeast phase. Conversion to the yeast phase was confirmed in all isolates (120/120): macroscopic evaluation revealed glabrous, cream-colored colonies, and microscopic examination of Panoptic-stained smears prepared from aliquots of these cultures revealed budding yeast cells and morphological forms consistent with the hypha-to-yeast transition. Phenotypic identification was based on the macroscopic and microscopic characteristics of the filamentous and yeast phases [30,31].

Cats were classified as culture-negative when no *Sporothrix* spp. growth was observed after up to 35 days of incubation at 25 °C. These animals were enrolled on clinical or epidemiological grounds (Section 2.1) and therefore represent suspected cases lacking laboratory confirmation, rather than disease-free animals. None had received antifungal therapy, including itraconazole, before sampling. Inadequate cytological or culture specimens and contaminated cultures were re-sampled whenever possible. When cytology was suggestive but no growth occurred, repeat sampling was requested to allow differentiation from other fungal pathogens, mainly *Histoplasma*. All owners were registered to receive results as promptly as possible. Culture-negative cats were assigned to a single mutually exclusive category according to their final classification: (i) contaminated culture for which re-sampling was not feasible; (ii) inadequate specimen for which re-sampling was requested but could not be obtained; (iii) alternative infectious or neoplastic diagnosis; and (iv) etiology undetermined.

The molecularly confirmed isolates (*n* = 37; 30.8% of the 120 culture-confirmed cases) were obtained between 2022 and 2026 and originated from 18 neighborhoods distributed across five sectors of the municipality: Northwest (Barra do Ceará, Vila Velha, Jardim Guanabara, and Jardim Iracema), West (Bom Jardim, Canindezinho, Planalto Ayrton Senna, and Henrique Jorge), Central (Fátima, Rodolfo Teófilo, Montese, and José Bonifácio), East (Aldeota and Dionísio Torres), and South/Southeast (Messejana, Jangurussu, Ancuri, and Conjunto Palmeiras). The annual distribution of tested isolates relative to the 120 confirmed cases is presented in Table S2. The tested isolates covered 2022 (2/2), 2025 (16/61; 26.2%), and 2026 (19/56; 33.9%) and originated from neighborhoods that account for 77 of the 113 confirmed cases with known neighborhoods (68.1%). No isolates were tested in 2023 (no cases) or in 2024 (one case, without neighborhood information), and some neighborhoods with high case counts, such as Mondubim and Paupina, were not represented. Species-level identification was therefore based exclusively on species-specific PCR applied to this subset of isolates; sequencing of the amplified products was not performed.

DNA was extracted from yeast-phase cultures, obtained as described above, for the isolates processed at the Molecular Biology Laboratory of the Federal Rural University of the Amazon (UFRA). A Promega DNA extraction kit (Promega Corporation, Madison, WI, USA) was used for 37 isolates following the manufacturer’s instructions.

Species-specific PCR targeting the calmodulin gene was performed using the primers Sbra-F (5′-CCCCCGTTTGACGCTTGG-3′) and Sbra-R (5′-CCCGGATAACCGTTGTGTCATAAT-3′). Each 20-μL reaction contained 5.5 μL of ultrapure water, 10 μL of Master Red master mix containing dNTPs, reaction buffer, Taq DNA polymerase, and MgCl_2_ (Ampliqon A/S, Odense, Denmark), 1 μL of each primer, and 2.5 μL of extracted DNA. Amplification consisted of initial denaturation at 95 °C for 5 min, followed by 35 cycles of denaturation at 94 °C for 30 s, annealing at 55 °C for 40 s, and extension at 72 °C for 30 s, with a final extension at 72 °C for 5 min. Reference strains of *S. brasiliensis*, obtained from the Evandro Chagas Institute (Belém, Pará, Brazil), were included in all assays as positive and specificity controls. Additionally, no-template controls (NTC) using ultrapure water and extraction blank controls were included in every PCR run to rule out contamination.

PCR products were analyzed by electrophoresis on a 1.5% agarose gel prepared in 1× TAE buffer and stained with GelGreen Nucleic Acid Stain (Biotium, Fremont, CA, USA). Each gel contained 0.375 g of agarose, 25 mL of 1× TAE buffer, and 2.5 μL of the 10,000× stain stock solution. Five microliters of each PCR product were loaded per well. Electrophoresis was performed at 90 V and 100 mA for 50 min, and the amplification products were visualized using an LED transilluminator (L-Pix Touch, Loccus Biotecnologia, Cotia, SP, Brazil).

### 2.4. Spatial Distribution

Human sporotrichosis notifications and culture-confirmed feline cases were aggregated by neighborhood and displayed using choropleth maps produced in QGIS software (version 4.0). Maps represented cumulative recorded case counts over the study period and, for feline cases, their distribution across the specified temporal intervals. Data for 2026 were restricted to January–March. Records without neighborhood information were excluded from neighborhood-level mapping. These maps describe the geographic distribution of recorded detections [32]. Therefore, the correspondence between human and feline cases can be assessed visually.

Additionally, to describe the agreement between feline and human records, the rates of confirmed cats and human notifications per 100,000 inhabitants (based on Census population) were calculated for each neighborhood, along with Spearman’s rank correlation coefficient between the number of confirmed cats and human notifications across the 116 neighborhoods. The 95% confidence interval was estimated using cluster bootstrap (percentile method; 5000 resamples; seed 12345; administrative regions as clusters). This analysis is exploratory, does not adjust for spatial dependence, and does not account for surveillance effort.

### 2.5. Statistical Analysis

Statistical analyses were conducted separately at the neighborhood and individual-animal levels (occurrence, for neighborhood-level: culture-confirmed cases versus no culture-confirmed cases; diagnostic, for individual-level: culture-confirmed versus culture-negative) [33].

Spearman’s rank correlation coefficient (ρ) was used to assess associations between the explanatory variables and the binary outcome at each analytical level [33]. Neighborhood-level analyses used the binary occurrence outcome rather than the case-count categories displayed on the maps. Individual-level analyses used explicitly defined binary contrasts, with coding and reference categories reported in Table S3. Records coded as “NI” (not informed) were excluded separately for each contrast. For rearing habits, the contrast compared exclusively indoor with exclusively outdoor cats; cats with mixed indoor–outdoor access were excluded from this specific comparison. Consequently, the number of observations could differ between contrasts.

Approximate 95% confidence intervals for ρ were estimated using 5000 cluster-bootstrap replicates, resampling administrative regions at the neighborhood level and neighborhoods at the individual level. Replicates in which the coefficient was undefined because either variable had no variation were excluded, and the number of valid replicates was recorded. Conventional two-sided Spearman *p*-values were adjusted using the Benjamini–Yekutieli procedure to control the false discovery rate within each prespecified family of comparisons [34]. These *p*-values were not adjusted for within-group dependence; multiplicity adjustment and cluster-bootstrap confidence intervals address different sources of uncertainty. Accordingly, correlations were interpreted as exploratory associations, considering their magnitude, confidence intervals, and adjusted *p*-values.

Logistic Regression and Random Forest models were fitted separately at each analytical level to predict the corresponding recorded binary outcome [21,22,35]. Individual-level analyses included an expanded dataset retaining “NI” as an explicit category and a complete-case sensitivity analysis; missing values were not imputed. Neighborhood-level analyses were additionally repeated after excluding human and animal health-service variables. Model development used nested, group-stratified cross-validation, with administrative regions defining groups for neighborhood-level models and neighborhoods defining groups for individual-level models. Independent variables were standardized using StandardScaler within the modeling pipelines, with the scaler fitted exclusively to the training data in each inner and outer fold [35,36]. Model tuning, performance assessment, uncertainty estimation, and SHAP interpretation are detailed in Section 2.6.

Lastly, Principal Component Analysis (PCA) was applied to map the variance structure of the data rather than for traditional dimensionality reduction purposes. PCA [37]. Exploratory PCA results, including explained variance and component loadings, are presented in the Supplementary Materials.

### 2.6. Predictive Machine Learning Modeling

Predictive modeling was conducted separately at the neighborhood and individual-animal levels, without combining predictors measured at different hierarchical scales. Analyses were exploratory and assessed the predictability of recorded surveillance outcomes rather than population infection risk. Predictor definitions, coding, and missing data proportions are provided in Table S3. Neighborhood-level findings were not interpreted as individual-level associations, and the modeled outcomes did not represent species-specific occurrence because molecular identification was available for only a subset of isolates.

At the neighborhood level, the outcome was the occurrence of at least one culture-confirmed feline sporotrichosis case during January 2022–March 2026. Of the 116 neighborhoods in the territorial database, 61 contributed at least one investigated cat and were included in modeling: 34 with and 27 without confirmed cases. Uninvestigated neighborhoods were excluded rather than classified as negative. Predictors were defined by the spatial and socio-environmental characteristics listed in Table S3. A sensitivity analysis excluded human and animal health-service variables because they may reflect access to investigation and case detection. It is worth noting that restricting the analysis to investigated neighborhoods did not fully account for differences in surveillance intensity.

At the individual level, fungal culture result was the outcome, and predictors were defined by the demographic, clinical, and epidemiological variables listed in Table S3. The expanded analysis included 239 cats from 61 neighborhoods: 113 culture-positive and 126 culture-negative animals. Seven additional culture-positive cats lacked neighborhood information and were retained only in the overall and temporal descriptions. Missing predictor information was represented by an explicit “NI” category, without imputation, and interpreted as a characteristic of record completeness rather than a biological risk factor. A complete-case sensitivity analysis included 120 cats from 47 neighborhoods, comprising 56 culture-positive and 64 culture-negative animals. This sensitivity analysis assessed dependence on missing information categories but did not eliminate potential selection bias associated with record completeness. Sample selection is presented in Figure S1.

Logistic Regression (LR) and Random Forest (RF) classifiers were evaluated using nested five-fold cross-validation, with StratifiedGroupKFold applied in both the inner and outer loops [22,35,36]. Grouping units were the 12 Regional Executive Secretariats (SER) for neighborhood-level analyses and neighborhoods for individual-level analyses. Group identifiers were excluded from the predictors. Reliable household identifiers were unavailable, preventing household-specific validation and estimation of the number of cats sampled per household. Neighborhood grouping prevented observations from the same neighborhood from appearing in both training and test partitions but did not directly quantify household dependence. Administrative grouping likewise did not guarantee spatial independence between groups, and residual spatial autocorrelation was not formally assessed.

Within each outer training partition, an inner five-fold grid search selected hyperparameters by minimizing mean validation logistic loss [38,39,40]. The selected pipeline was then fitted to the outer training data and evaluated on the held-out fold. LR and RF used identical outer partitions within each analytical scenario. Categorical predictors were dummy encoded, and StandardScaler was fitted exclusively within the training partitions of both cross-validation loops [41]. The LR grid evaluated L1, L2, Elastic Net, and unpenalized configurations where supported by the solver [42]; the RF grid evaluated tree-complexity and feature-selection settings. Both models considered balanced and unweighted classes. No resampling of outcome classes or decision-threshold optimization was performed. Search grids and hyperparameters selected in each outer fold are reported in the Supplementary Material.

Performance metrics comprised accuracy, balanced accuracy, macro-averaged precision and F1-score, ROC-AUC, PR-AUC, and the Brier score. In this binary task, balanced accuracy equals macro-averaged recall. PR-AUC was calculated by trapezoidal integration, whereas the Brier score summarized probabilistic prediction error and was not interpreted as an isolated measure of calibration. Fold-specific metrics were reported as means and standard deviations; these standard deviations describe between-fold variability and were not interpreted as confidence intervals [43,44].

ROC-AUC and PR-AUC were also calculated from pooled out-of-fold (OOF) probabilities, with each observation predicted by a model trained without its test group. Pooled OOF estimates were distinguished from mean fold-specific AUCs, since these summaries are not interchangeable. Approximate 95% confidence intervals were obtained from 5000 cluster-bootstrap replicates, resampling SER or neighborhoods, as appropriate, and retaining all observations within each selected group. Replicates containing only one outcome class were excluded, and confidence limits were defined by the 2.5th and 97.5th percentiles. Pointwise percentile bands for ROC and precision–recall curves were derived from the same procedure. Therefore, these were not simultaneous confidence bands. Because models were not refitted during bootstrapping, these intervals are conditional on the OOF predictions and do not capture the full variability of model development. Interpretation also considered the small number of groups, particularly at the neighborhood level. Outcome prevalence, majority-class accuracy, and ROC-AUC = 0.5 were reported as reference benchmarks separately for each analytical sample. Model comparisons were descriptive; no paired superiority test or repeated nested cross-validation was performed.

Model interpretation used Shapley Additive Explanations (SHAP) [45]. Feature-ranking stability was evaluated on each held-out outer fold using its corresponding fitted pipeline and a permutation explainer of positive-class probability, with up to 100 background observations sampled exclusively from the outer training partition. Features were ranked by mean absolute SHAP value within each fold. Stability summaries comprised median and minimum–maximum ranks and the frequency of inclusion among the three highest-ranked features, retaining ties at the cutoff and excluding zero-importance features. Rankings referred to the encoded features, including individual dummy variables where applicable, and were not aggregated automatically into their original categorical variables. These summaries describe variability across the five observed folds rather than uncertainty under repeated model fitting. They were not treated as confidence intervals or evidence that highly ranked predictors had statistically significant associations with the outcome.

After performance evaluation, final models were tuned by grouped cross-validation and fitted to each complete analytical dataset for descriptive SHAP beeswarm plots. These plots were distinguished from the held-out-fold stability analysis. Complete-case analyses provided SHAP results without missing information indicators. SHAP contributions were interpreted as explanations of model predictions, not causal effects. Their interpretation was considered alongside predictive performance: a prominent or stable SHAP ranking did not establish predictive utility, and disagreement between SHAP rankings and Spearman correlations was not, by itself, considered evidence of nonlinear effects or interactions. Neither the full-data refits nor their SHAP plots constituted independent validation.

Analyses used seed 12345 for cross-validation, cluster bootstrapping, and RF fitting, and 12345 plus the fold number for fold-specific SHAP calculations. Implementation used Google Colaboratory and Jupyter notebooks with Python 3.13.16, scikit-learn 1.6.1, pandas 2.2.3, NumPy 2.1.3, and SHAP 0.54.1 [45,46,47,48].

## 3. Results

Between January 2022 and March 2026, a total of 246 felines presenting with clinical suspicion of sporotrichosis were evaluated across 116 neighborhoods in the municipality of Fortaleza, Ceará. Out of these, 120 animals received laboratory confirmation. Cytological smears identified yeast-like structures compatible with *Sporothrix* spp. in 99 felines, whereas fungal culture was positive in all 120 confirmed cases, and all isolates showed hypha-to-yeast conversion at 37 °C. All cytologically positive animals were concurrently confirmed via mycological culture. For definitive molecular identification, 37 isolates were selected, and 100% (37/37) were successfully identified as *Sporothrix brasiliensis* using species-specific PCR targeting the CAL gene. The number of animals and neighborhoods included in each analysis is shown in Figure S1.

Culture was negative in 126 of the 246 cats investigated (51.2%). Among these, 15 (11.9%) could not be re-sampled despite the request, owing to culture contamination (*n* = 9) or inadequate specimens (*n* = 6; sample decomposition, *n* = 3; delayed transport, *n* = 2; adherent slides, *n* = 1). An alternative diagnosis was established in 26 cats (20.6%), either infectious (*n* = 19; *Histoplasma* spp., *n* = 3; *Microsporum canis*, *n* = 8; *Trichophyton* spp., *n* = 1; *Cryptococcus* spp., *n* = 3; *Leishmania* spp., *n* = 1; *Candida* spp., *n* = 2; and *Malassezia* spp., *n* = 1) or neoplastic (*n* = 7; squamous cell carcinoma). The etiology remained undetermined in 85 cats (67.5%) (Table S4). All cats with cytology compatible with sporotrichosis yielded positive cultures.

**Figure 1:**
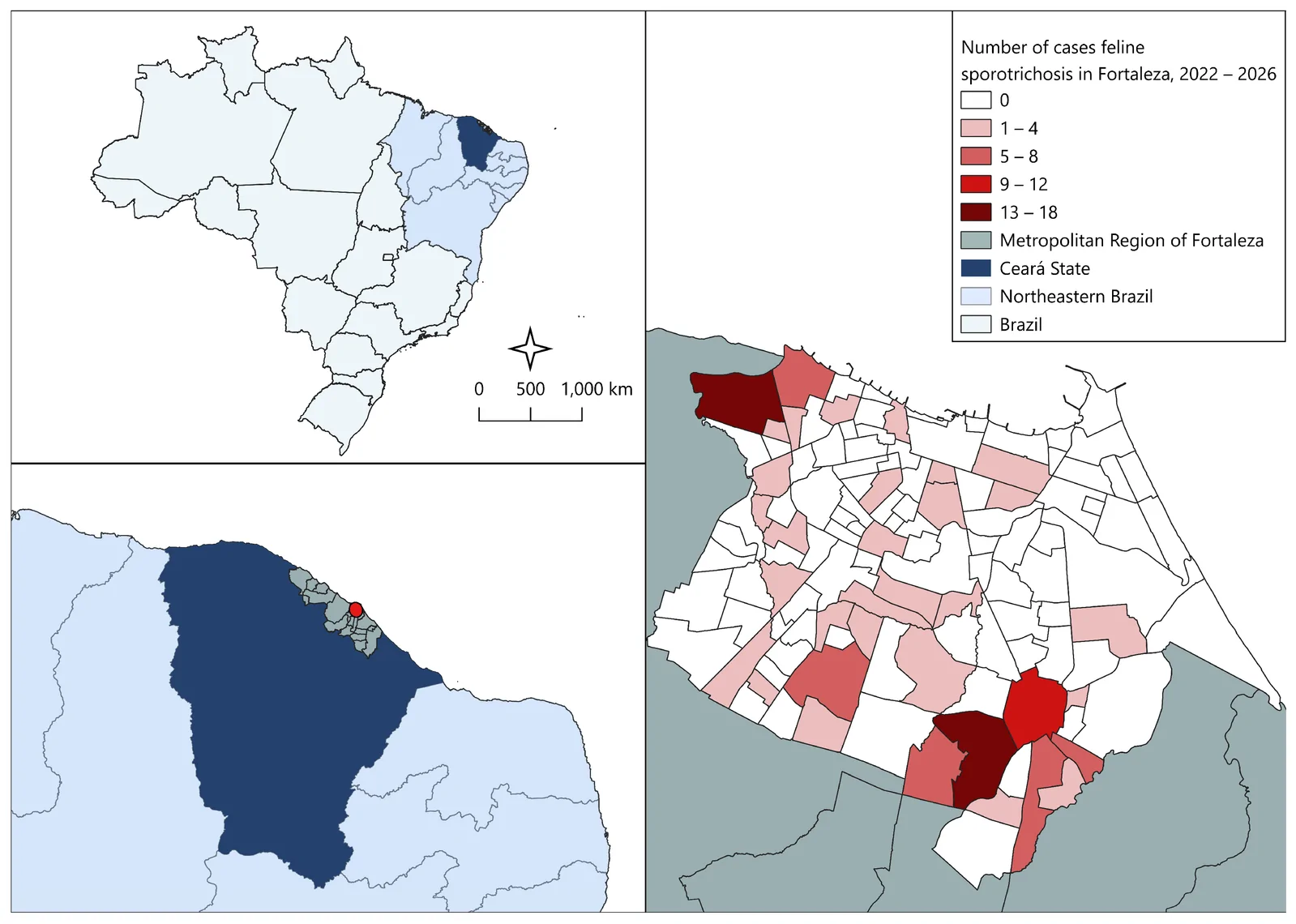
Geographic distribution of cumulative culture-confirmed feline sporotrichosis case counts by neighborhood in Fortaleza, Ceará, Brazil (January 2022–March 2026). The red point indicates the city of Fortaleza.

Of the 246 suspected cats investigated, neighborhood information was available for 239 (97.2%), distributed across 61 neighborhoods. The remaining 7 cats (2.8%), all of them culture-confirmed, lacked neighborhood information owing to incomplete address data; they were included in the overall and temporal analyses but excluded from the spatial analyses. Of the 116 neighborhoods, 61 (52.6%) were represented by at least one clinically suspected cat. Among these, 34 (55.7%) had at least one culture-confirmed case, corresponding to 29.3% of the 116 standardized study units; these analyses were based on the 113 confirmed cases with an identifiable neighborhood. The highest numbers of confirmed cases were recorded in Vila Velha (*n* = 18; 15.0% of all 120 confirmed cases) and Jangurussu (*n* = 17; 14.2%), followed by Messejana (*n* = 9; 7.5%) and Mondubim (*n* = 8; 6.7%). Neighborhood-level data (population, investigated cats, confirmed cats, and human notifications) are available in Table S5. The cumulative spatial distribution of recorded cases from January 2022 through March 2026 showed higher concentrations of detected cases in the southeastern, western, and southern areas of the municipality (Figure 1).

Among the 120 culture-positive cats, unneutered males were the most frequent group (*n* = 36; 30.0%), and most animals were adults (*n* = 105; 87.5%). Clinically, the disseminated form was the most frequent (*n* = 54; 45.0%), followed by the localized cutaneous form (*n* = 35; 29.2%), with the lesion description missing for 31 cats (25.8%). Notably, one asymptomatic cat, included because it cohabited with a positive animal, had no visible skin lesions but tested positive by fungal culture from a nasal mucosal swab. In the models, this animal was classified as “lesion distribution not reported.” Regarding the rearing system, 67 cats (55.8%) were semi-domiciled or free-roaming and 43 (35.8%) were kept strictly indoors, with information missing for 10 (8.3%). The distributions of sex, neutering status, age, rearing system, animal hoarding, and exposure, including missing data, for culture-positive and culture-negative cats are presented in Tables S5 and S6. The proportion of missing data differed between groups, being higher among culture-positive cats for animal hoarding (34.2% vs. 19.0% among culture-negative cats) and exposure (15.8% vs. 4.0%). The mean time elapsed between initial lesion onset and veterinary care at the UVZ was 88 days (median = 66 days).

**Figure 2:**
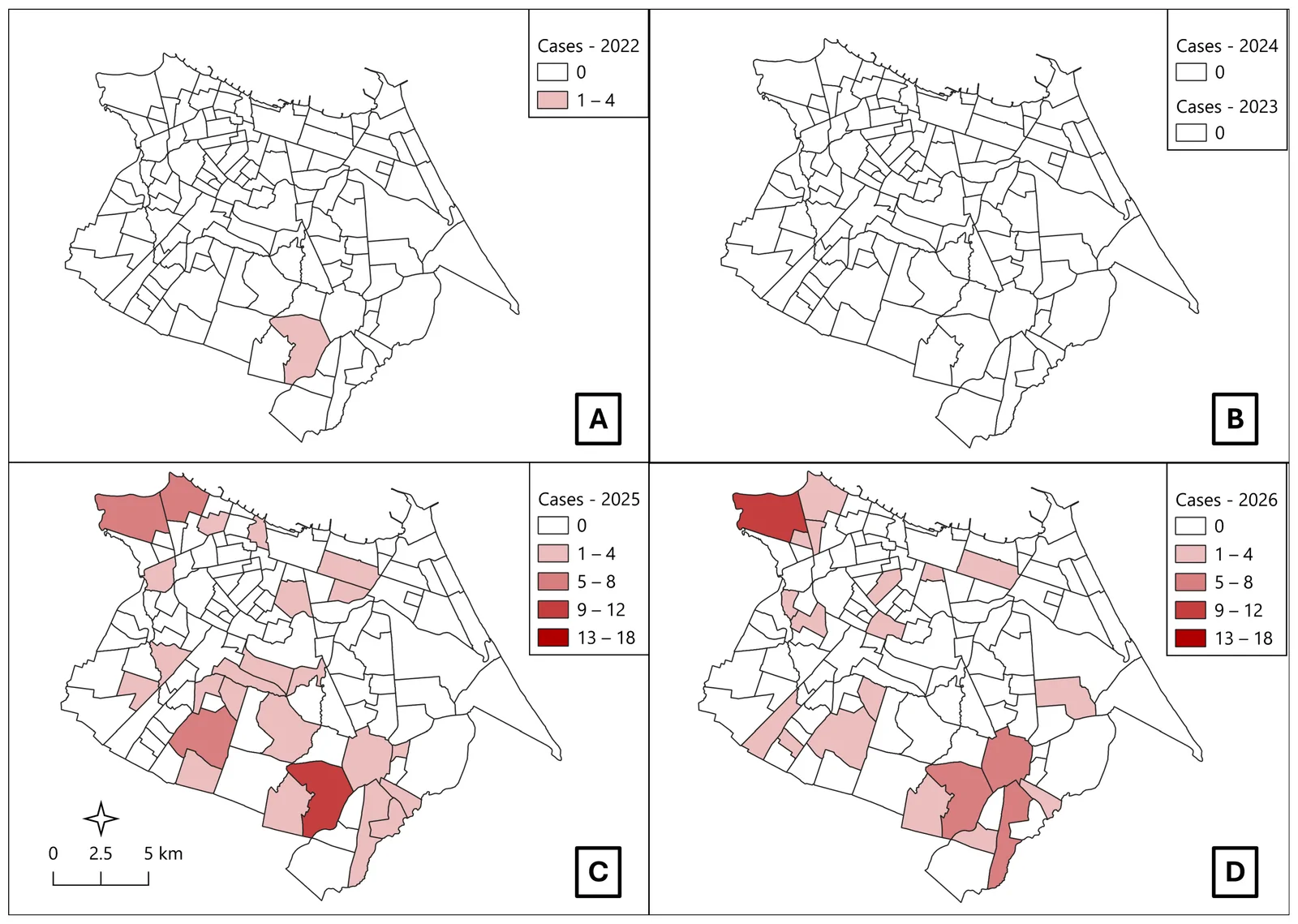
Geographic distribution of culture-confirmed feline cases by period. (**A**) 2022 (*n* = 2). (**B**) 2023–2024: no cases could be mapped (no cases in 2023; the single case in 2024 lacked neighborhood information). (**C**) 2025 (*n* = 57 mapped of 61; 4 without neighborhood information). (**D**) January–March 2026 (*n* = 54 mapped of 56; 2 without neighborhood information).

The number of culture-confirmed feline cases recorded was 2 in 2022, 0 in 2023, 1 in 2024, 61 in 2025, and 56 between January and March 2026, totaling 120 cases, of which 113 had neighborhood information and were mapped (Figure 2).

Counts for 2022–2025 represent complete calendar years, whereas the 2026 count covers only the first quarter. The first recorded cases (*n* = 2) were confirmed in 2022 (Figure 2A), both originating from the Jangurussu neighborhood, followed by an absence of confirmed cases recorded through public passive surveillance during 2023 and one case in 2024, which had no neighborhood information and therefore could not be mapped (Figure 2B). However, in 2025, an increase in the number of recorded cases was observed, with 61 cases, 57 of which could be assigned to a neighborhood (4 lacked neighborhood information), led by Jangurussu (*n* = 10), Barra do Ceará (*n* = 6), Mondubim (*n* = 6), and Vila Velha (*n* = 6) (Figure 2C). Between January and March 2026 alone, 56 cases had already been confirmed (54 with neighborhood information and 2 without), heavily concentrated in Vila Velha (*n* = 12), Messejana (*n* = 6), Paupina (*n* = 5), and Jangurussu (*n* = 5) (Figure 2D).

From January 2022 to March 2026, 120 of the 246 cats investigated were culture-positive, corresponding to an overall laboratory confirmation proportion of 48.8% (95% Wilson CI: 42.6–55.0%). The proportion of culture confirmation was 60.4% in 2025 (61/101) and 39.4% in January–March 2026 (56/142), a period in which systematic active case finding was implemented and the number of cats investigated increased (Table S7).

Laboratory-confirmed feline cases were initially detected in one neighborhood in 2022, representing a cumulative territorial coverage of 0.9% (1/116). No additional neighborhoods recorded a first detection in 2023 or 2024. In 2025, first detections occurred in 23 additional neighborhoods, increasing the cumulative number of neighborhoods with recorded cases to 24 and the cumulative territorial coverage to 20.7% (24/116). Between January and March 2026, 10 additional neighborhoods recorded their first laboratory-confirmed case, resulting in a cumulative total of 34 neighborhoods and a territorial coverage of 29.3% (34/116) by the end of the study period.

The mean monthly number of neighborhoods with a first recorded culture-confirmed feline case was 1.9 in 2025 (23/12) and 3.3 during January–March 2026 (10/3).

**Figure 3:**
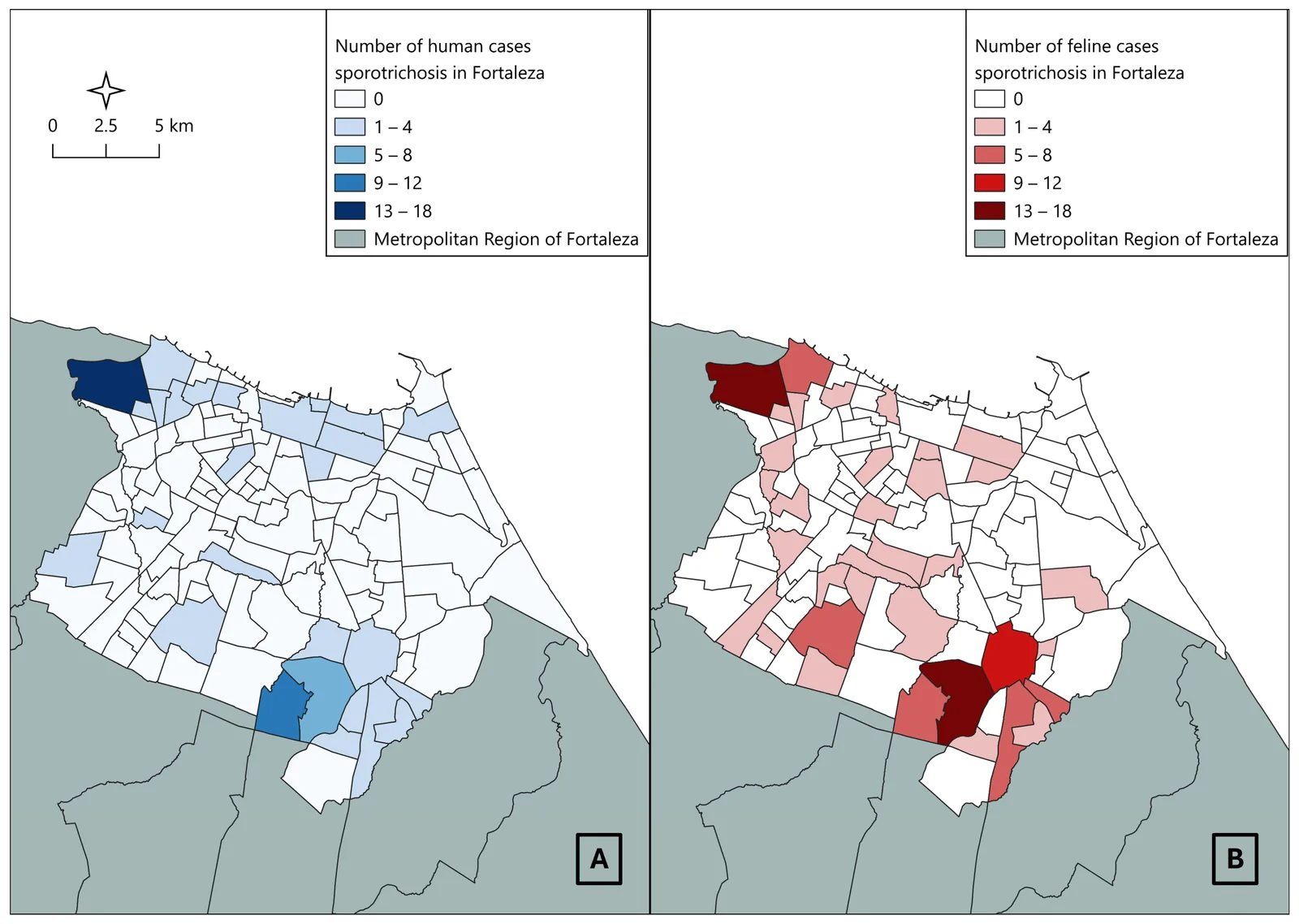
Geographic distribution of cumulative human sporotrichosis notifications (**A**) and culture-confirmed feline cases (**B**) by neighborhood in Fortaleza, January 2022–March 2026.

Human notifications extracted from SIMDA revealed a temporal pattern overlapping with the feline epizootic, shifting from zero cases in 2022 and 2023 to two initial human cases in 2024 located in Messejana and Jangurussu. In 2025, human notifications spiked to 55 cases distributed across 23 neighborhoods, predominantly in Vila Velha (*n* = 15), Conjunto Palmeiras (*n* = 7), and Jangurussu (*n* = 5), followed by 11 human cases registered across eight neighborhoods in the first quarter of 2026, totaling 68 human cases. The integration of the maps showed partial correspondence between human notifications (Figure 3A) and feline cases (Figure 3B). The seven neighborhoods with the highest counts of feline cases (6 to 18 cases: Vila Velha, Jangurussu, Messejana, Mondubim, Barra do Ceará, Conjunto Palmeiras, and Paupina) all recorded human notifications (2 to 18).

Overall, human notifications were recorded in 25 of the 116 neighborhoods, and 16 of them also had confirmed cats. Among the 34 neighborhoods with confirmed cats, 16 (47%) had human notifications, and 57 of the 68 notifications (84%) occurred in neighborhoods with confirmed cats. The number of confirmed cats was correlated with the number of human notifications (ρ = 0.51; 95% CI 0.19 to 0.71). Conversely, nine neighborhoods had human notifications without confirmed feline cases (e.g., Barroso and Centro, with 2 each). Citywide, the rates were 4.8 confirmed cats and 2.9 human notifications per 100,000 inhabitants. Among neighborhoods with 20,000 or more inhabitants, the highest rates of confirmed cats were observed in Vila Velha (34.0), Conjunto Palmeiras (24.8), and Jangurussu (24.1), and the highest rates of human notifications in Vila Velha (34.0) and Conjunto Palmeiras (31.9) (Table S6). This correspondence is descriptive, based on absolute counts and rates per 100,000 inhabitants, and does not account for spatial dependence or surveillance effort.

The spatial distribution of feline sporotrichosis occurrences revealed marked territorial contrasts associated with local socio-environmental indicators. As detailed in Table 1, five of the six neighborhoods with the highest recorded counts (Vila Velha, Jangurussu, Conjunto Palmeiras, Mondubim, and Barra do Ceará) presented the maximum score on the precarious settlements scale (Level 5), associated with low Income-HDI values (0.01 to 0.08); the sixth, Messejana, was classified as Level 2 (Income-HDI 0.12). Furthermore, the overall distribution of investigated and positive cats according to these precarious settlement levels is summarized in Table 2. Additionally, four of these six neighborhoods had no registered animal health service units in the survey. In contrast, the four neighborhoods without confirmed cases showed lower levels of housing precariousness (Levels 0–1) and generally higher Income-HDI values (0.10–0.89, excluding Aracapé due to missing data), although overlapping ranges occurred. Because only 1 to 4 suspected cats were investigated in each of these lower-count neighborhoods, the absence of confirmed cases should not be interpreted as absence of the disease.

Exploratory Spearman analyses were conducted separately for the neighborhood-level and individual-level datasets. For the evaluated predictor–outcome contrasts, the group-bootstrap 95% confidence intervals included zero, and no null hypothesis was rejected after Benjamini–Yekutieli adjustment within the specified testing families. These findings did not establish the absence of associations. Correlation coefficients, confidence intervals, conventional *p*-values, BY-adjusted *p*-values, and the corresponding contrasts and sample sizes are presented in Table S8.

**Figure 4:**
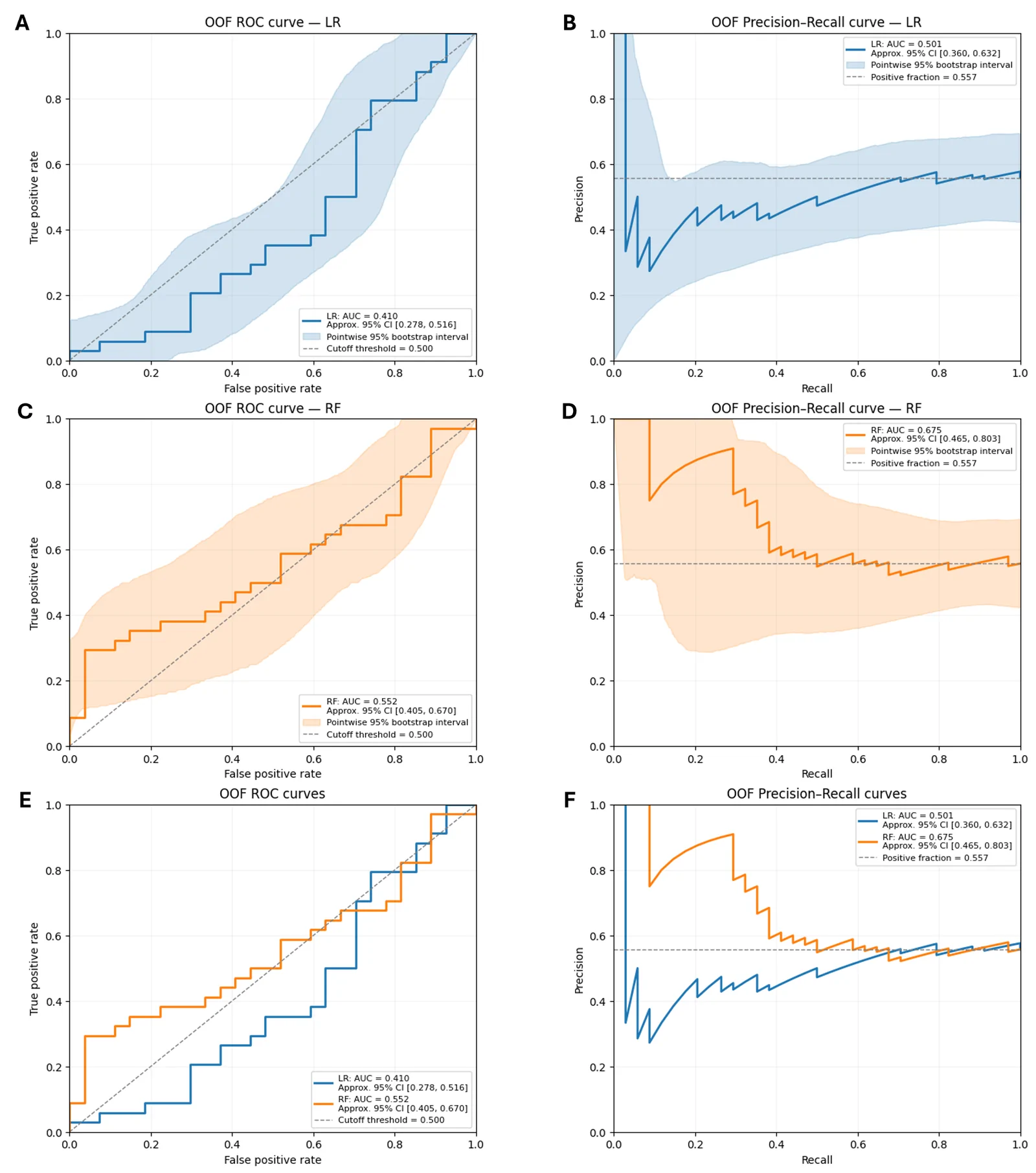
Receiver operating characteristic (ROC) and precision–recall (PR) curves for out-of-fold neighborhood-level model predictions for the ungrouped validation. (**A**) Out-of-fold (OOF) Receiver Operating Characteristic (ROC) curve for the Logistic Regression (LR) model; (**B**) OOF Precision-Recall (PR) curve for the LR model; (**C**) OOF ROC curve for the Random Forest (RF) model; (**D**) OOF PR curve for the RF model; (**E**) Combined OOF ROC curves for both LR and RF models; (**F**) Combined OOF Precision-Recall curves for both LR and RF models. The shaded areas represent the pointwise 95% bootstrap confidence intervals. In the PR curves (**B**,**D**,**F**), the horizontal dashed line indicates the baseline positive fraction of the sample (0.557). In the ROC curves (**A**,**C**,**E**), the diagonal dashed line represents the performance of a random classifier.

**Figure 5:**
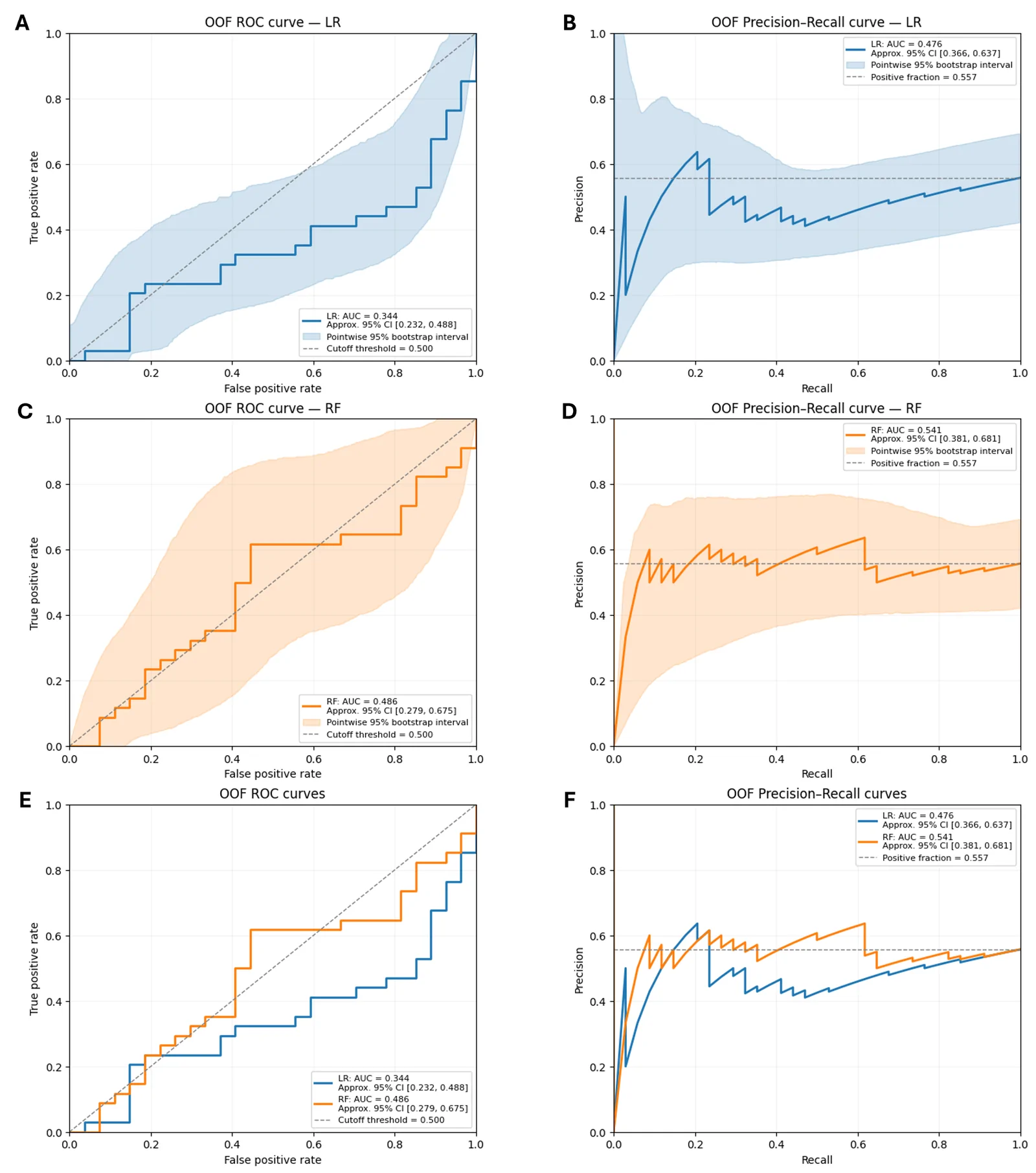
Receiver operating characteristic (ROC) and precision–recall (PR) curves for out-of-fold neighborhood-level model predictions for the grouped validation. (**A**) Out-of-fold (OOF) Receiver Operating Characteristic (ROC) curve for the Logistic Regression (LR) model; (**B**) OOF Precision-Recall (PR) curve for the LR model; (**C**) OOF ROC curve for the Random Forest (RF) model; (**D**) OOF PR curve for the RF model; (**E**) Combined OOF ROC curves for both LR and RF models; (**F**) Combined OOF Precision-Recall curves for both LR and RF models. The shaded areas represent the pointwise 95% bootstrap confidence intervals. In the PR curves (**B**,**D**,**F**), the horizontal dashed line indicates the baseline positive fraction of the sample (0.557). In the ROC curves (**A**,**C**,**E**), the diagonal dashed line represents the performance of a random classifier.

Predictive performance under grouped nested cross-validation is summarized in Table 3 and Table 4. In the neighborhood-level sample, 34 of the 61 investigated units were positive (55.7%), corresponding to a majority-class accuracy reference of 55.7%. With all predictors included, pooled OOF ROC-AUC was 0.344 (95% CI: 0.232–0.488) for LR and 0.486 (95% CI: 0.279–0.675) for RF. The corresponding pooled OOF PR-AUC values were 0.476 (95% CI: 0.366–0.637) and 0.541 (95% CI: 0.381–0.681), respectively. Mean fold-specific balanced accuracy was 33.77% ± 7.35% for LR and 57.14% ± 28.07% for RF. Mean fold-specific Brier scores were 0.356 and 0.274, respectively. The information for the grouped and ungrouped results is displayed in Figure 4 and Figure 5.

In the sensitivity analysis excluding human and animal health service predictors, pooled OOF ROC-AUC was 0.337 (95% CI: 0.210–0.484) for LR and 0.500 (95% CI: 0.285–0.723) for RF. Corresponding pooled OOF PR-AUC values were 0.461 (95% CI: 0.354–0.610) and 0.579 (95% CI: 0.390–0.779) (Table 3).

**Figure 6:**
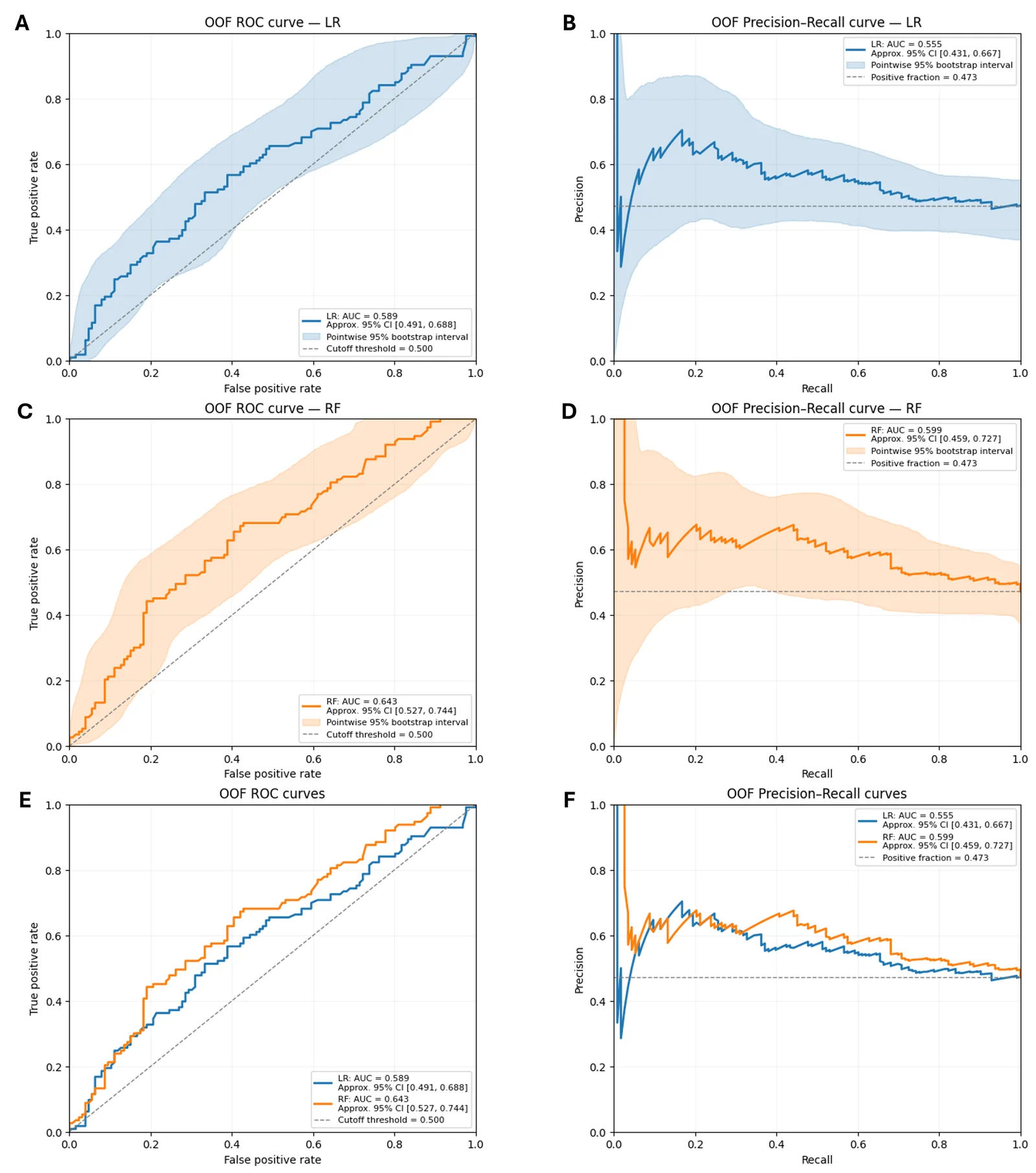
Out-of-fold ROC and precision–recall curves for cat-level model predictions, ungrouped validation. (**A**) Out-of-fold (OOF) Receiver Operating Characteristic (ROC) curve for the Logistic Regression (LR) model; (**B**) OOF Precision-Recall (PR) curve for the LR model; (**C**) OOF ROC curve for the Random Forest (RF) model; (**D**) OOF PR curve for the RF model; (**E**) Combined OOF ROC curves for both LRand RF models; (**F**) Combined OOF Precision-Recall curves for both LR and RF models. The shaded areas represent the pointwise 95% bootstrap confidence intervals. In the PR curves (**B**,**D**,**F**), the horizontal dashed line indicates the baseline positive fraction of the sample (0.473). In the ROC curves (**A**,**C**,**E**), the diagonal dashed line represents the performance of a random classifier.

**Figure 7:**
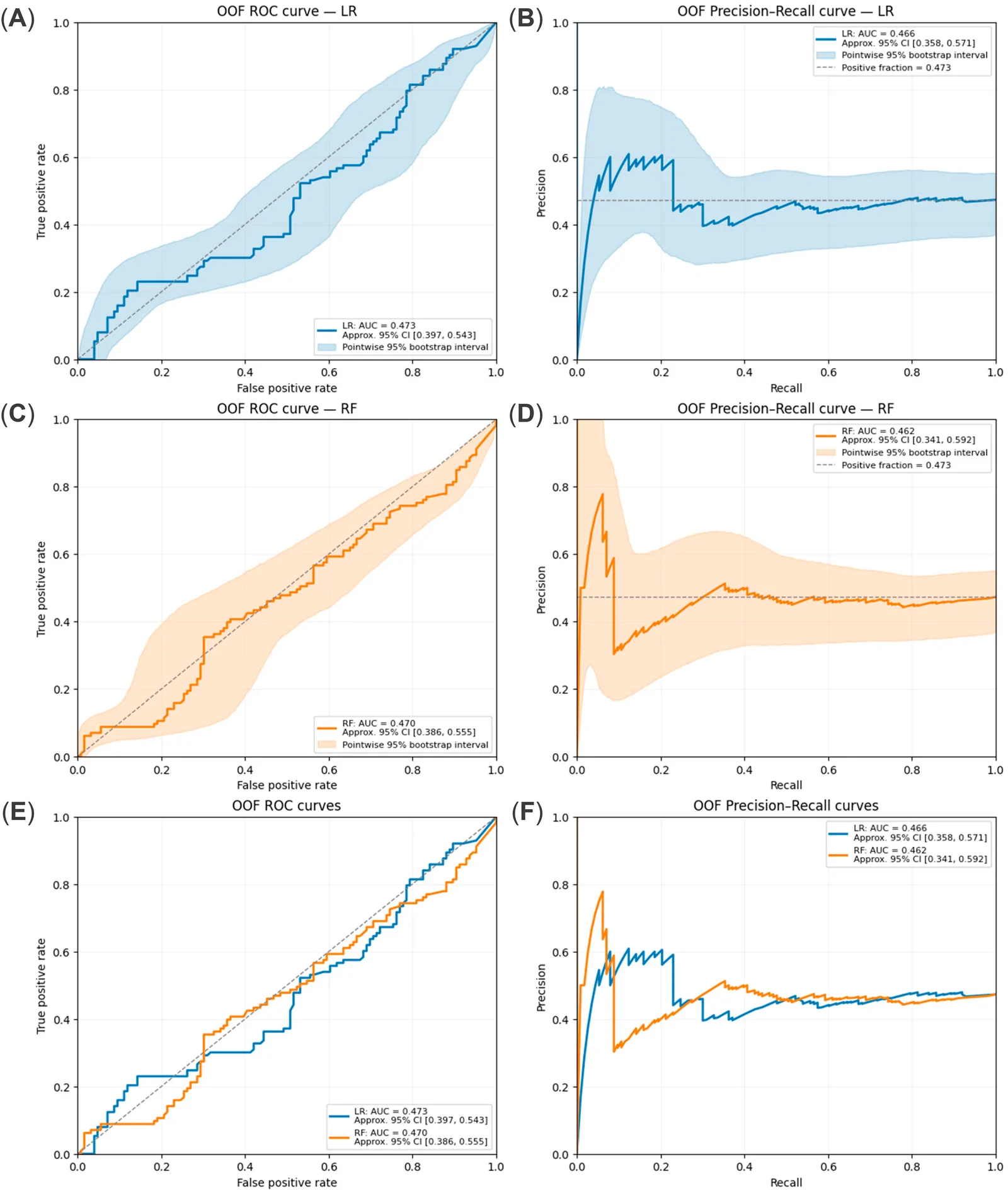
Out-of-fold ROC and precision–recall curves for cat-level model predictions grouped validation. (**A**) Out-of-fold (OOF) Receiver Operating Characteristic (ROC) curve for the Logistic Regression (LR) model; (**B**) OOF Precision-Recall (PR) curve for the LR model; (**C**) OOF ROC curve for the Random Forest (RF) model; (**D**) OOF PR curve for the RF model; (**E**) Combined OOF ROC curves for both LR and RF models; (**F**) Combined OOF Precision-Recall curves for both LR and RF models. The shaded areas represent the pointwise 95% bootstrap confidence intervals. In the PR curves (**B**,**D**,**F**), the horizontal dashed line indicates the baseline positive fraction of the sample (0.473). In the ROC curves (**A**,**C**,**E**), the diagonal dashed line represents the performance of a random classifier.

In the expanded individual-level sample, 113 of 239 cats were culture-positive (47.3%), and the majority-class accuracy reference was 52.7%. Pooled OOF ROC-AUC was 0.473 (95% CI: 0.397–0.543) for LR and 0.470 (95% CI: 0.386–0.555) for RF. Corresponding pooled OOF PR-AUC values were 0.466 (95% CI: 0.358–0.571) and 0.462 (95% CI: 0.341–0.592). Mean fold-specific balanced accuracy was 48.44% ± 5.52% for LR and 49.50% ± 4.77% for RF, with mean fold-specific Brier scores of 0.313 and 0.267, respectively. The information for the grouped and ungrouped results is displayed in Figure 6 and Figure 7.

**Table 1:** Epidemiological and socio-environmental profile of neighborhoods with the highest and lowest recorded counts of feline sporotrichosis, Fortaleza, January 2022–March 2026.

Neighborhood	Investigated Suspect Cats	Human Notifications	Confirmed Feline Cases	Demographic Density (pop/km^2^) *	Population (*n*)	HDI-Income	Precarious Settlements (Level 0–5) **	Vegetation Cover (m^2^/pop)	Health Services (Animal/Human)
Higher Recorded Counts									
Vila Velha	32	18	18	7396	52,881	0.07	5	54.32	Yes/4 units
Jangurussu	22	6	17	8819	70,651	0.05	5	19.35	No/4 units
Messejana	16	2	9	7137	43,205	0.12	2	15.18	Yes/3 units
Mondubim	14	2	8	8757	62,440	0.08	5	16.01	No/2 units
Barra do Ceará	12	2	7	14,824	63,477	0.05	5	4.33	No/4 units
Conj. Palmeiras	10	9	7	5135	28,223	0.01	5	59.60	No/2 units
Low/No Recorded Counts									
Panamericano	1	0	0	15,543	8580	0.10	0	0.84	No/1 unit
Cocó	1	0	0	7035	23,005	0.89	0	63.47	No/0 units
Maraponga	4	0	0	7347	12,482	0.20	1	18.74	No/0 units
Aracapé	2	0	0	8693	18,943	ND	1	7.80	No/4 units

* Inhabitants per km^2^; source (density and population): IBGE, 2022 Census. ** Precarious Settlements Index: Level 0 (formal area with complete infrastructure) to Level 5 (severe informal settlement lacking adequate sanitation, housing, and services). HDI-Income: income component of the HDI (Census); source: Fortaleza em Bairros (Fortaleza City Hall). Cases and notifications: cumulative from January 2022 to March 2026 (human cases from 2024 to 2026 recorded counts: the six neighborhoods with the most confirmed cats (culture-positive). Low/no recorded counts: neighborhoods without confirmed cases, with a precariousness level of 0–1, ranked by the number of suspect cats investigated (tie-breaker: lowest level). ND: no data available (Aracapé has no income data from the 2010 Census in the source).

**Table 2:** Distribution of investigated and positive cats for feline sporotrichosis according to the precarious settlement levels of neighborhoods in Fortaleza.

Precarious Settlement Level	Neighborhoods (*n*)	Neighborhoods with Confirmed Cases, *n* (%)	Investigated Cats	Positive Cats, *n* (%)	Median Investigated Cats per Neighborhood
0	4	2 (50%)	21	3 (14.3%)	2
1	26	11 (42%)	50	19 (38.0%)	1
2	11	9 (82%)	47	26 (55.3%)	2
3	5	3 (60%)	12	3 (25.0%)	3
4	5	2 (40%)	12	3 (25.0%)	1
5	10	7 (70%)	97	59 (60.8%)	6
Total	61	34 (56%)	239	113 (47.3%)	–

**Table 3:** Performance metrics of neighborhood-level Logistic Regression and Random Forest models across cross-validation folds and out-of-bag predictions.

Metric	LogReg (Random Folds, All Predictors)	RF (Random Folds, All Predictors)	Grouped LogReg (All Predictors)	Grouped RF (All Predictors)	LogReg	RF
Accuracy(%)	42.44 ± 9.40	50.64 ± 19.04	34.75% ± 8.25	55.88% ± 24.45	37.85% ± 11.91	55.88% ± 22.70
Balanced accuracy/Recall (macro) (%)	44.43 ± 9.59	50.19 ± 16.17	33.77 ± 7.35	57.14 ± 28.07	38.94 ± 15.63	54.40 ± 25.03
Precision (macro) (%)	43.41 ± 11.03	53.42 ± 22.41	33.75 ± 5.59	58.44 ± 23.84	32.62 ± 16.07	57.09 ± 21.51
F1-score (macro) (%)	40.94 ± 9.70	47.35 ± 17.16	30.66 ± 5.94	54.31 ± 24.75	33.03 ± 14.16	52.26 ± 22.28
ROC-AUC (mean across folds)	0.44 ± 0.14	0.57 ± 0.26	0.37 ± 0.16	0.49 ± 0.28	0.37 ± 0.24	0.56 ± 0.30
PR-AUC (mean across folds)	0.56% ± 0.06	0.65 ± 0.19	0.58 ± 0.15	0.56 ± 0.23	0.56 ± 0.13	0.66 ± 0.23
OOF ROC-AUC [95% CI]	0.410 [0.278–0.516]	0.552 [0.405–0.670]	0.344 [0.232–0.488]	0.486 [0.279–0.675]	0.337 [0.210–0.484]	0.500 [0.285–0.723]
OOF PR-AUC [95% CI] (baseline 0.557)	0.501 [0.360–0.632]	0.675 [0.465–0.803]	0.476 [0.366–0.637]	0.541 [0.381–0.681]	0.461 [0.354–0.610]	0.579 [0.390–0.779]
Brier score (mean across folds; baseline 0.247)	0.322	0.248	0.356	0.274	0.336	0.268

LogReg: Logistic Regression; RF: Random Forest; ROC-AUC: Receiver Operating Characteristic-Area Under the Curve; PR-AUC: Precision-Recall Area Under the Curve; OOF: Out-of-fold; CI: Confidence Interval. Values presented as mean ± standard deviation or estimate [95% CI].

**Table 4:** Predictive performance of individual-level machine learning models comparing the full sample (*n* = 239) and complete-case analysis (*n* = 120).

Metric	LogReg (Random Folds, Full Sample)	RF (Random Folds, Full Sample)	Grouped LogReg (Full Sample)	Grouped RF (Full Sample)	Grouped LogReg (Complete-Case)	Grouped RF (Complete-Case)
Accuracy (%)	58.99 ± 3.56	62.32 ± 5.09	47.87% ± 5.99	48.42% ± 5.70	38.06% ± 5.27	46.35% ± 7.76
Balanced accuracy/Recall (macro) (%)	58.60 ± 3.65	61.95 ± 4.72	48.44 ± 5.52	49.50 ± 4.77	37.53 ± 5.02	46.49 ± 8.41
Precision (macro) (%)	58.81 ± 3.68	64.02 ± 8.09	48.43 ± 5.58	49.72 ± 4.81	39.37 ± 4.00	45.31 ± 9.41
F1-score (macro) (%)	58.60 ± 3.699	61.31 ± 4.11	47.41 ± 5.89	47.90 ± 5.50	35.90 ± 4.57	40.78 ± 8.02
ROC-AUC (mean across folds)	0.59 ± 0.09	0.65 ± 0.07	0.45 ± 0.06	0.45 ± 0.07	0.37 ± 0.05	0.41 ± 0.14
PR-AUC (mean across folds)	0.57 ± 0.09	0.65 ± 0.10	0.50 ± 0.14	0.49 ± 0.15	0.37 ± 0.21	0.35 ± 0.20
OOF ROC-AUC [95% CI]	0.589 [0.491–0.688]	0.643 [0.527–0.744]	0.473 [0.397–0.543]	0.470 [0.386–0.555]	0.384 [0.277–0.503]	0.376 [0.276–0.492]
OOF PR-AUC [95% CI] (baseline: full sample 0.473; complete-case 0.467)	0.555 [0.430–0.667]	0.599 [0.459–0.727]	0.466 [0.358–0.571]	0.462 [0.341–0.592]	0.402 [0.289–0.517]	0.384 [0.275–0.482]
Brier score (mean across folds; baseline 0.249)	0.261	0.233	0.313	0.267	0.305	0.283

LogReg: Logistic Regression; RF: Random Forest; ROC-AUC: Receiver Operating Characteristic-Area Under the Curve; PR-AUC: Precision-Recall Area Under the Curve; OOF: Out-of-fold; CI: Confidence Interval. Values presented as mean ± standard deviation or estimate [95% CI].

The complete-case analysis included 120 cats, of which 56 were culture-positive (46.7%), corresponding to a majority-class accuracy reference of 53.3%. Pooled OOF ROC-AUC was 0.384 (95% CI: 0.277–0.503) for LR and 0.376 (95% CI: 0.276–0.492) for RF. Corresponding pooled OOF PR-AUC values were 0.402 (95% CI: 0.289–0.517) and 0.384 (95% CI: 0.275–0.482) (Table 4).

Overall, the evaluated models did not demonstrate meaningful discrimination under grouped validation in the available analytical samples. Results from random-fold validation are presented separately as complementary analyses in Table 3 and Table 4. Model comparisons were descriptive; no formal paired test of superiority was performed.

**Figure 8:**
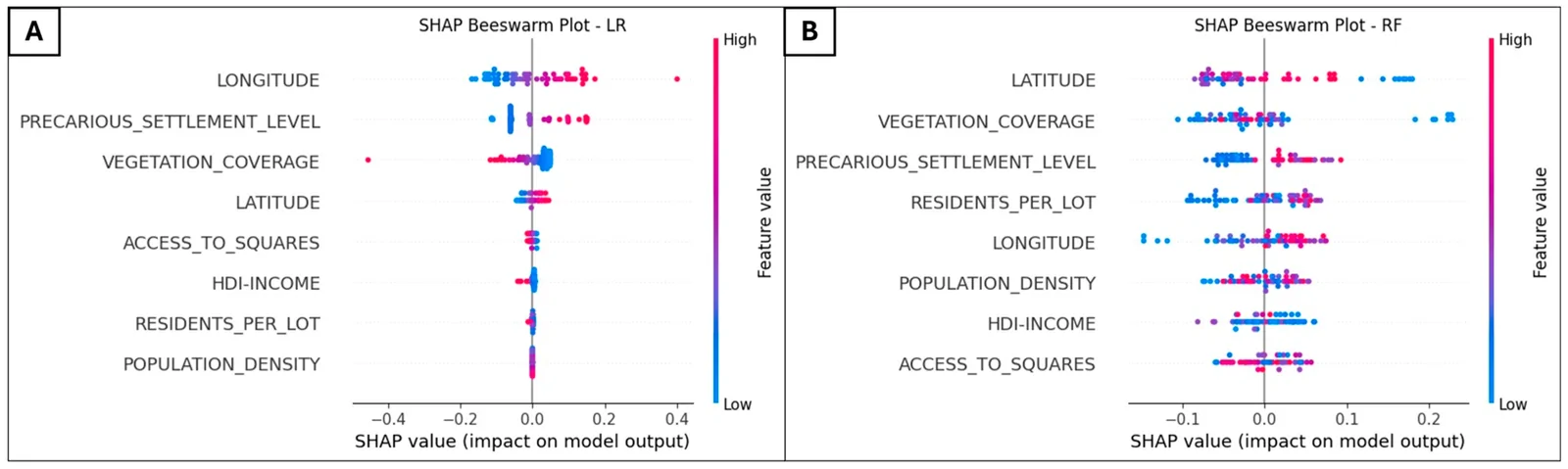
SHAP beeswarm plots for the neighborhood-level models: (**A**) binary logistic regression and (**B**) Random Forest. Predictors are ranked by mean absolute SHAP values. Each point represents a neighborhood, and its horizontal position indicates the predictor’s contribution to the model output. Colors indicate predictor values, from low (blue) to high (red).

**Figure 9:**
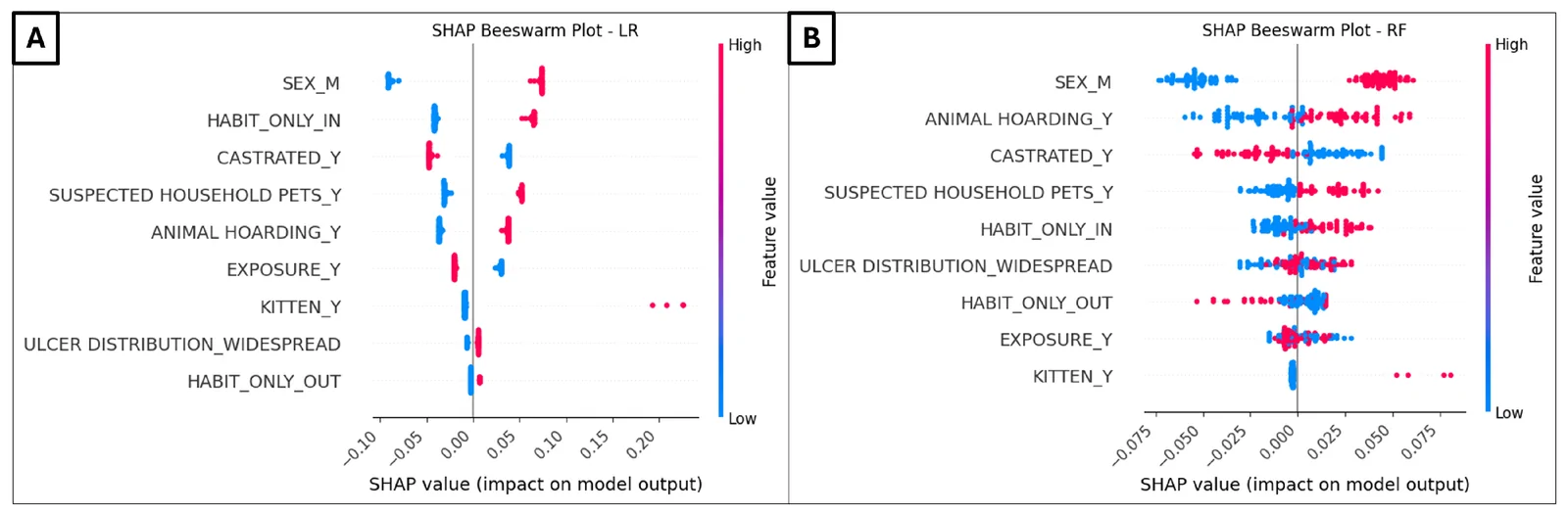
SHAP beeswarm plots for the individual animal-level models: (**A**) binary logistic regression and (**B**) Random Forest. Predictors are ranked by mean absolute SHAP values. Each point represents a cat, and its horizontal position indicates the predictor’s contribution to the model output. Colors indicate predictor values, from low (blue) to high (red). For binary indicators, these values correspond to 0 and 1, respectively.

SHAP beeswarm plots describe contributions to the predicted probability of culture-confirmed occurrence for the models fitted to the sensitivity analysis neighborhood-level and the complete-case individual-level analytical datasets (Figure 8 and Figure 9). These data explanations were considered separately from predictive performance and from the stability analysis based on held-out outer folds.

At the neighborhood-level, precarious settlement level had a median rank of 2 (range, [1,2,3,4,5]) and appeared among the three highest-ranked features in 3/5 folds for LR model excluding health service predictors. At the individual level, rankings refer to encoded indicators rather than aggregated original variables. At the complete-case setting, masculine sex had a median rank of 1 (range, 1–7) and appeared among the three highest-ranked features in 4/5 folds for both models. Complete rank summaries for the ablation settings are presented in Table S9A–D.

In addition, three-dimensional projections of the PCA for both neighborhood-level in Figure S2A,B and individual animal-level predictors in Figure S3A,B demonstrated a scattered distribution and substantial overlap between outcome classes. Detailed component loadings and variance contributions for both predictor sets are presented in Table S10.

## 4. Discussion

This study represents the first systematic evaluation of the feline sporotrichosis epidemic in Fortaleza, from its first documented cases to its establishment as a major urban health crisis. It also exploratorily evaluates whether available socio-environmental and individual variables can predict disease occurrence. All 37 isolates subjected to species-specific PCR were identified as *Sporothrix brasiliensis* (37/37; 100%). These isolates represented 30.8% of the 120 culture-confirmed feline cases (37/120). Therefore, species-level identification was restricted to this subset, and the findings cannot be extrapolated to the untested isolates. This finding helps challenge the classical view of sporotrichosis as a predominantly sapronotic event linked exclusively to soil contact in rural environments, establishing it as an aggressive, urban, and animal-driven zoonosis within the municipality [1,4]. The presence of *S. brasiliensis* among the molecularly tested isolates may help explain the abrupt transition from an isolated index case in 2022 to an increasing geographic distribution of recorded detections by early 2026. This species is widely recognized as the most virulent member of the *Sporothrix* genus, exhibiting high zoonotic potential due to the high fungal load sustained in feline cutaneous and mucosal lesions, which considerably facilitates cross-species transmission even during superficial contact [49,50].

The high frequency of the disseminated clinical presentation (45%) observed in Fortaleza closely mirrors the patterns of hyperendemicity previously documented in Rio de Janeiro and, more recently, in Pernambuco and Paraíba [6,8]. This clinical pattern does not establish intrinsic hypervirulence of the local fungal lineage. The mean interval between the first observation of lesions and veterinary assessment at the UVZ was 88 days. One hypothesis is that a prolonged interval before assessment could allow clinical progression and extend opportunities for transmission. However, the relationship between this interval and clinical presentation was not analyzed in this study. Reverse causation is also possible, as more extensive lesions may prompt earlier veterinary consultation. Therefore, the available data do not establish whether the observed interval contributed to the frequency of disseminated disease or to further transmission.

The temporal and spatial distribution of recorded cases must be interpreted considering the modifications in the municipal surveillance framework. The absence of confirmed cases in 2023 and the registration of only one case in 2024, following the initial confirmations in 2022, likely reflect limitations in early clinical recognition and the exclusive reliance on passive surveillance, rather than an epidemiological interruption of pathogen circulation.

During this initial period, cases were restricted to a single neighborhood in 2022, representing 0.9% of the municipality’s neighborhoods (1/116), with no detections in new areas during 2023 and 2024. Conversely, a marked increase in both case volume and geographic distribution was documented from 2025 onward, coinciding with the gradual implementation of active surveillance strategies. The localized active case finding initiated in 2025 in response to community demand revealed 61 cases and expanded first detections to 23 additional neighborhoods, increasing the cumulative territorial coverage to 20.7% (24/116). The subsequent systematic active search by endemic disease control agents in early 2026 further enhanced the sensitivity of the municipal diagnostic workflow: between January and March alone, 56 cases were confirmed and another 10 neighborhoods were added, bringing the coverage to 29.3% (34/116). Therefore, this progression reflects both the biological dissemination and establishment of *S. brasiliensis* in the urban matrix, and the methodological shifts in case recognition and investigation driven by the active surveillance efforts.

By documenting the increasing number of neighborhoods with laboratory-confirmed cases, this study provides evidence to characterize the municipal distribution of recorded sporotrichosis detections and inform surveillance priorities. The results also show that, with the available variables, predictive modeling did not demonstrate meaningful discrimination under grouped validation. Recommendations from the broader literature support expanding access to diagnosis and antifungal treatment, alongside owner education, responsible animal management, and population control and neutering strategies, as components of an integrated response to disease emergence [3,4,49]. The effectiveness of these interventions was not evaluated in this study.

The statistical and machine learning analyses provided uncertain estimates of the relationship between feline sporotrichosis and the evaluated socio-environmental conditions, which diverges from findings reported in other epidemiological studies [50]. For the evaluated predictor–outcome contrasts, no null hypothesis was rejected after Benjamini–Yekutieli correction within the specified testing families, and the group-bootstrap confidence intervals included zero. These findings do not establish the absence of associations and should be distinguished from the descriptive correlation between human notification counts and confirmed feline case counts. Under grouped validation, the models failed to achieve meaningful discrimination at both levels of analysis, underscoring the difficulty of capturing the disease complexity, at least under the conditions of the present study. Neighborhoods such as Jangurussu, Vila Velha, and Conjunto Palmeiras concentrated more recorded cases within contexts of precarious settlements and socioeconomic disadvantage (Table 1); this descriptive observation, based on a small number of neighborhoods, provides a useful indicator to guide surveillance but does not allow for inferring causal effects. A plausible explanation for these results is that case detection was linked to surveillance intensity, which expanded throughout the study (passive until 2024, localized active searching in 2025, and systematic active searching in 2026). Restricting the analysis to investigated neighborhoods avoided classifying uninvestigated areas as negative, but did not account for differences in investigation intensity among included neighborhoods; some neighborhoods without confirmed cases had few investigated cats. The limited sample size (61 neighborhoods and 12 regions) and number of events also constrain the precision of the estimates, possibly restricting statistical power to detect moderate associations. These findings support expanding feline investigation, recording surveillance effort and more detailed exposure information, and analyzing a larger number of neighborhoods.

Overall, the Spearman coefficients indicated weak or negligible estimated monotonic associations, with uncertainty around their magnitude and direction. These results do not exclude nonlinear, nonmonotonic, or predictor-dependent relationships [51,52]. However, such relationships were not demonstrated by the correlation results or SHAP importance rankings. Socio-environmental conditions, including sanitation and waste management, remain hypotheses for further investigation. Environmental contamination, rodent abundance, cat aggregation, and fungal persistence in organic material were not measured in this study, and SHAP contributions do not establish these mechanisms. In accordance with the principles of peripheral One Health, urban sporotrichosis should not be viewed solely as an isolated infectious disease, but must be considered within the broader context of socio-spatial inequality and access to public services, even though a direct causal link to transmission was not established in the present sample [17,53,54].

At the neighborhood level, 55.7% of the investigated units had confirmed cases, resulting in a relatively balanced class distribution. Pooled OOF ROC-AUC values were 0.344 for LR and 0.486 for RF with all predictors included; the corresponding values in the expanded individual-level sample were 0.473 and 0.470 (Table 3 and Table 4). These results did not demonstrate relevant discrimination under grouped validation. Model comparisons were descriptive and did not establish statistically demonstrated superiority of RF over LR. The limited discrimination may reflect sample size, ascertainment, measurement quality, and incomplete predictor information, but their individual contributions were not quantified. The bootstrap intervals were conditional on fixed OOF predictions and did not capture the full uncertainty of model development. Further development and independent validation are therefore required before these models can support operational surveillance decisions.

SHAP explanations should be interpreted alongside this limited predictive performance. The beeswarm plots describe contributions to positive-class probabilities from models fitted to the ablation settings, whereas the outer-fold analysis evaluates variability in feature rankings across held-out samples. A prominent or stable ranking does not establish predictive utility, statistical significance, or a causal effect. Differences between SHAP rankings and Spearman coefficients do not, by themselves, demonstrate nonlinear relationships or interactions.

Unneutered males accounted for 30.0% of culture-confirmed cases. Of the 120 culture-positive cats, 43 (35.8%) were reported as strictly indoor. These descriptive proportions do not quantify the risk associated with sex, neutering status, or rearing system, and the occurrence of confirmed cases among indoor cats does not establish a specific exposure route for indoor cats. Possible explanations include unreported outdoor access, contact with infected animals [1,50], or indirect exposure through contaminated materials; none of these pathways was demonstrated in this study. As no environmental sampling was performed, the findings do not establish contamination within or around the affected households or its contribution to infection. These data also do not allow the protective effect of indoor confinement to be quantified.

A positive nasal culture was obtained from an asymptomatic cat cohabiting with an affected animal. This finding raises the possibility of subclinical infection or carriage, but a single positive culture cannot distinguish these explanations from transient colonization or contamination of the sampled mucosa. It also does not establish persistent carriage, infectiousness, or a reservoir role for this animal. Although viable fungi have been isolated from nasal samples and droplets expelled during sneezing in clinically affected cats [55], this evidence does not establish the same shedding capacity in the asymptomatic animal identified in this study.

This finding may inform future studies involving longitudinal clinical and mycological monitoring of contact cats, including apparently healthy animals, to investigate persistent culture positivity, the development of clinical signs, and their potential role in transmission. These investigations could also assess the utility of cytological or mycological screening of contact animals and the integration of findings into surveillance and epidemiological mapping systems.

The overlap between feline and human records is a recurrent descriptive finding, with most human notifications (84%) occurring in neighborhoods with confirmed cats, and the highest counts standardized per 100,000 human inhabitants in both groups observed in Vila Velha and Conjunto Palmeiras among neighborhoods with 20,000 or more inhabitants (Figure 3; Table S6). This correspondence is consistent with the role of cats as a source of human infection in sporotrichosis caused by *S. brasiliensis* [1,2,3,4], but does not establish transmission links between the individual human and feline cases recorded in this study. It supports the joint assessment of human and animal surveillance data to identify priority areas for further epidemiological investigation [4,17,18]. The finding also points to concrete opportunities: nine neighborhoods had human notifications without confirmed feline cases, and in five of them no cat was investigated (Carlito Pamplona, Floresta, Joaquim Távora, João XXIII, and Praia de Iracema), indicating where feline investigation could be expanded.

This analysis is descriptive. Population-standardized counts can be unstable in small neighborhoods, the detection of cats and the notification of human cases depend on surveillance intensity, and spatial autocorrelation was not assessed, so the correlation neither implies causation nor quantifies risk. Feline detections expressed per 100,000 human inhabitants do not estimate feline incidence. Future studies should incorporate surveillance effort and appropriate population denominators, and account for spatial dependence when evaluating geographic associations between human and feline cases [4,17,18].

In Messejana, 5 of the 9 confirmed cats were semi-domiciled or free-roaming and 4 were kept strictly indoors; no rearing-system data was missing. However, these data do not establish whether specific feline behaviors or local patterns of human–animal interaction explain the difference between the number of confirmed feline cases (*n* = 9) and human notifications (*n* = 2), because they do not characterize the frequency or nature of contact between infected cats and residents. Six of the nine feline cases in this neighborhood were confirmed in the first quarter of 2026, but the temporal relationship between these detections and human infections was not evaluated. Differences in case detection (active and passive surveillance for cats, predominantly passive surveillance for humans) and possible human underreporting may also contribute, but these explanations were not evaluated specifically in Messejana. Therefore, the lower number of human notifications should not be interpreted as evidence of reduced zoonotic risk. Investigations of affected households, integrating human and animal surveillance data, could help clarify this pattern.

Finally, human sporotrichosis notifications recorded in SIMDA averaged 4.6 cases per month in 2025 (55 cases over 12 months) and 3.7 cases per month during January–March 2026 (11 cases over three months). During the latter period, 56 feline cases were confirmed, averaging 18.7 cases per month. These monthly counts provide a common temporal scale but remain subject to differences in human and animal surveillance. These findings reinforce the importance of timely data sharing between human and veterinary health services, improved diagnostic recognition, and integrated surveillance to clarify temporal patterns and support coordinated responses to emerging sporotrichosis, including the procurement and distribution of antifungal medications for human and animal populations [18].

For human sporotrichosis, itraconazole and lipid formulations of amphotericin B, the latter indicated for severe or disseminated disease, are provided free of charge through the Brazilian Unified Health System (SUS) [56]. For feline sporotrichosis, itraconazole, alone or combined with potassium iodide, is a recommended therapeutic option [3]. Where feline treatment costs are borne by owners, financial constraints may limit treatment access and continuity. Treatment discontinuation for financial reasons has been documented in feline cases elsewhere in Brazil [57]. However, individual treatment use, adherence, outcomes, and treatment-related costs were not assessed in this study, and municipal funding was not analyzed. Therefore, the contribution of financial barriers or treatment access to the observed increase in reported cases could not be determined.

Descriptive characterization indicates that several neighborhoods with the highest recorded feline case counts had characteristics of substantial territorial vulnerability. Five of the six neighborhoods with the highest case counts fall into the highest precarious settlement category (Level 5) with Income-HDI ranging from 0.01 to 0.08 (Table 1), and Level 5 neighborhoods combined accounted for approximately half of all confirmed cases (59/113) and 40.6% of investigated cats with neighborhood information (97/239) descriptions of cat-transmitted sporotrichosis in low-income urban settings [17,53,54] and supports further investigation of housing precariousness as a potential correlate of recorded occurrence.

However, the data do not establish a direct causal relationship or an independent risk factor: there was no monotonic gradient across precariousness levels, and the confidence interval for Spearman’s rank correlation (ρ = 0.18; 95% CI −0.06 to 0.42) included zero. The difference disappeared among neighborhoods with two or more investigated cats, indicating that surveillance and investigation intensity may contribute to the observed spatial concentration, but their contribution was not quantified.

Nevertheless, the observed convergence between areas with higher case registration and the highest level of the precarious settlements scale (Level 5), while remaining descriptive, indicates the importance of considering socio-environmental determinants when interpreting the local dynamics of this zoonosis. Recorded cases, gaps in investigation, and barriers to diagnostic access can inform further territorial assessment, but these analyses do not establish a validated ranking of neighborhood risk. Further hypotheses may be drawn regarding the convergence of lower income, scarce public veterinary infrastructure, and high disease reporting, suggesting these as key indicators for prioritizing targeted territorial interventions. In this context, integrated territorial actions aligned with the One Health perspective may offer a promising approach. Coordinating basic veterinary care, such as diagnostic access, surveillance, and neutering campaigns, with broader efforts to address housing and sanitation in the most deprived areas (Levels 4 and 5) may be considered within this framework [3,4,17,53,54] as a strategy to optimize the allocation of public resources and strengthen health surveillance where risk and vulnerability overlap. However, the effectiveness of these interventions and their optimal spatial allocation were not evaluated in this study.

## 5. Limitations

This study has limitations, which should be read in light of its context: the first recorded cases in the study dataset occurred in 2022, and the number of recorded cases increased rapidly, so data collection, diagnostic workflows, financial resources, and government support had to be created and adapted during the outbreak itself. First, the progressive expansion of the affected territory and of surveillance (passive until 2024, localized active case finding in 2025, and systematic in 2026) made it difficult to keep data uniform across periods and neighborhoods and may have altered detection; surveillance effort was not quantified, and feline population denominators were unavailable, precluding estimation of feline incidence and adjustment for investigation effort. Only neighborhoods with suspected cats entered the models, since investigated neighborhoods without confirmed cases should not be interpreted as disease-free. Second, it is worth noting the considerable amount of missing data, which may not have been missing at random, although the missingness mechanism was not established: 7 of the 246 cats (2.8%) had no neighborhood information; among the 239 cats with neighborhood information, 36.8% had no information on suspected household animals and 4.2% on habit; the proportion of missing data differed between positive and negative cats in the full sample of 246 cats (animal hoarding, 34.2% versus 19.0%; exposure, 15.8% versus 4.0%; Table S5), which may reflect how the information was recorded rather than biological differences. Data were not imputed, and missing categorical values were labeled as NI. The complete-case analysis reduced the sample to 120 cats, about half of the total, and may have introduced additional selection bias. Reliable household identifiers and the number of households were unavailable, preventing assessment of within-household dependence.

Third, the socio-environmental variables were measured at the neighborhood level and cannot be attributed to individual cats or households (ecological fallacy) [23], and observations from neighboring neighborhoods or cats from the same neighborhood may be dependent: validation was grouped by administrative region and by neighborhood, respectively, but administrative grouping does not ensure spatial independence, and residual spatial autocorrelation was not assessed. Conventional Spearman *p*-values did not account for within-group dependence; multiplicity adjustment does not address this limitation.

Fourth, culture, although the reference standard, has imperfect sensitivity: in 67.5% of cats classified as culture-negative, no alternative etiology was identified, so some of them may represent false negatives; these animals were clinically suspected cases, not healthy controls, and the direction and magnitude of any bias arising from outcome misclassification remain uncertain. Even though samples were obtained across a diverse range of neighborhoods, molecular identification was restricted to 37 of the 120 culture-positive isolates (30.8%); therefore, species identification cannot be extrapolated to untested isolates.

Fifth, the number of events was small (34 positive neighborhoods, 12 administrative regions for validation, and 10 original predictor variables; 113 positive cats for 8 original predictor variables, so the estimates may be unstable, particularly given the small number of territorial groups. All selected penalized LR fits used C = 10, the smallest tested C value and therefore the strongest regularization among the penalized configurations in the reported grid. Bootstrap intervals for pooled OOF AUCs were conditional on fixed predictions and therefore did not fully capture model-development uncertainty. SHAP attribution summaries do not establish causal effects, and their stability does not demonstrate predictive utility. The modeling results should therefore be considered exploratory, and future studies, with more resources and data standardized from the start, may lessen these limitations.

## 6. Conclusions

This study presents the first systematic characterization of feline sporotrichosis in Fortaleza and documents the increase in recorded cases and the geographic expansion of detections between January 2022 and March 2026, from one to 34 neighborhoods. *Sporothrix brasiliensis* was identified in all 37 molecularly tested samples, representing 30.8% of the 120 positive cultures; this finding confirms the presence of the species among the analyzed isolates but cannot be extrapolated to untested isolates or used to estimate the rate of zoonotic transmission. The neighborhoods with the highest recorded case counts were concentrated in contexts of greater housing precariousness, even though an independent association was not established. The role of housing conditions remains a hypothesis to be tested. In the exploratory predictive analyses, with grouped cross-validation and correction for multiple testing, the available socio-environmental and individual variables did not demonstrate better than chance discrimination of the recorded outcomes. Separately, none of the predictor–outcome associations met the statistical significance criterion after correction for multiple testing within the specified families of comparisons. This result is compatible with a complex, multifactorial disease. However, these findings do not establish the reasons for the limited predictive performance, highlighting the need for more complete data, larger samples, additional epidemiologically relevant predictors, and independent validation before these models can support operational decisions.

The findings reinforce the importance of strengthening integrated human and animal health surveillance, standardized case investigation, and access to diagnosis and treatment, within a One Health approach. Although the effectiveness of specific control interventions was not evaluated in this study, the literature supports the hypothesis that the outbreak response may include cat neutering, decentralized and free diagnosis, and access to prolonged antifungal treatment, in addition to sanitation improvements in the most affected peripheral areas [3,4,49]. Given the expanding geographic distribution of its recorded cases, zoonotic sporotrichosis warrants continued public health attention in Fortaleza, underscoring the need to recognize it as a public health priority among neglected tropical diseases in Northeastern Brazil. The data gathered here provide a descriptive basis for further investigation and surveillance planning. The identified limitations reflect the recent emergence of recorded cases in the municipality, where sporotrichosis was not reported before 2022, and reinforce the need for government support and funding to standardize data collection and strengthen surveillance as the disease expands.

## Data Availability

The original contributions presented in this study are included in the article/supplementary material. Further inquiries can be directed to the corresponding author(s).

## Supplementary Materials

The following supporting information can be downloaded at: https://www.mdpi.com/article/10.3390/ani16213498/s1, Table S1: Official neighborhoods of Fortaleza merged into a single row in the database (CSV), with the row identification and the criterion indicating the merge; Table S2: Temporal and geographic distribution of isolates tested by species-specific PCR relative to culture-confirmed feline cases (*n* = 120), Fortaleza, January 2022 to March 2026; Table S3: Variables used in the neighborhood-level and animal-level models: definition, coding, and percentage of missing data. Missing data were kept as a “not reported” category and were not imputed; Figure S1: Flow of animals and neighborhoods in each analysis; Table S4: Classification of the 126 culture-negative cats; Table S5: Investigated (suspected) cats, culture-confirmed cats, human sporotrichosis notifications, and population by neighborhood in Fortaleza (*n* = 116), January 2022 to March 2026; Table S6: Characteristics of the cats investigated according to culture result, Fortaleza, January 2022 to March 2026 (*n* = 246); Table S7A: Cats investigated, culture-confirmed cases, and surveillance strategy by period, Fortaleza, January 2022 to March 2026; Table S7B: Selected hyperparameters in each outer fold of nested cross-validation; Table S8: Exploratory Spearman rank correlations, 95% cluster-bootstrap confidence intervals, and Benjamini–Yekutieli multiplicity-adjusted *p*-values between socio-environmental and individual predictors and feline sporotrichosis detection outcomes in Fortaleza, Brazil; Table S9A: SHAP Feature Stability—Neighborhood Level (Logistic Regression); Table S9B: SHAP Feature Stability—Neighborhood Level (Radom Forest); Table S9C: SHAP Feature Stability—Individual Level (Logistic Regression); Table S9D: SHAP Feature Stability—Individual Level (Radom Forest); Figure S2: Principal component analysis of neighborhood-level predictors; Figure S3: Principal component analysis of individual animal-level predictors; Table S10: Principal Component Analysis (PCA) loadings and proportion of explained variance for the first three principal components (PC1–PC3) across individual-level and neighborhood-level predictor sets.

## Abbreviations

The following abbreviations are used in this manuscript:

Abbreviation	Full Term/Definition
AUC-ROC	Area Under the Receiver Operating Characteristic Curve
BHI	Brain Heart Infusion
BLR	Binary Logistic Regression
bp	Base Pair
CAL	Calmodulin (gene)
CC BY	Creative Commons Attribution License
CEUA	Institutional Ethics Committee on Animal Use
CI	Confidence Interval
DNA	Deoxyribonucleic Acid
dNTPs	Deoxynucleoside Triphosphates
F1-score	F-measure/F-score (macro-averaged)
FDR	False Discovery Rate
HDI-Income	Human Development Index-Income Component
IBGE	Brazilian Institute of Geography and Statistics
LCP	Laboratory Confirmation Proportion
LISA	Local Indicators of Spatial Association
LR	Logistic Regression
N	Population Size/Sample Size
NI/NR	Not Informed/Not Reported
NTC	No-Template Control
OOF	Out-Of-Fold
OR	Odds Ratio
PC	Principal Component
PCA	Principal Component Analysis
PCR	Polymerase Chain Reaction
PR-AUC	Area Under the Precision-Recall Curve
RF	Random Forest
ROC	Receiver Operating Characteristic
Sbra-F	*Sporothrix brasiliensis* Forward (primer)
Sbra-R	*Sporothrix brasiliensis* Reverse (primer)
SER	Regional Executive Secretariat
SHAP	Shapley Additive Explanations
SIMDA	Daily Disease Monitoring System
SUS	Unified Health System (Brazilian)
TAE	Tris-Acetate-EDTA
TC	Territorial Coverage
UECE	State University of Ceará
UFRA	Federal Rural University of the Amazon
UV	Ultraviolet
UVZ	Zoonosis Surveillance Unit

## References

[1] Rodrigues A.M., Gonçalves S.S., de Carvalho J.A., Borba-Santos L.P., Rozental S., Camargo Z.P. (2022). Current progress on epidemiology, diagnosis, and treatment of sporotrichosis and their future trends. J. Fungi.

[2] Almeida-Paes R., de Oliveira M.M.E., Freitas D.F.S., do Valle A.C.F., Zancopé-Oliveira R.M., Gutierrez-Galhardo M.C. (2014). Sporotrichosis in Rio de Janeiro, Brazil: *Sporothrix brasiliensis* Is associated with atypical clinical presentations. PLoS Negl. Trop. Dis..

[3] Gremião I.D.F., Rocha E.M.S., Montenegro H., Carneiro A.J.B., Xavier M.O., Farias M.R., Monti F., Mansho W., Pereira R.H.M.A., Pereira S.A. (2021). Guideline for the management of feline sporotrichosis caused by *Sporothrix brasiliensis* and literature revision. Braz. J. Microbiol..

[4] Gremião I.D.F., Menezes R.C., Schubach T.M.P., Figueiredo A.B.F., Cavalcanti M.C.H., Pereira S.A. (2015). Feline sporotrichosis: Epidemiological and clinical aspects. Med. Mycol..

[5] Rabello V.B.S., Almeida M.A., Bernardes-Engemann A.R., Almeida-Paes R., Macedo P.M., Zancopé-Oliveira R.M. (2022). The historical burden of sporotrichosis in brazil: A systematic review of cases reported from 1907 to 2020. Braz. J. Microbiol..

[6] Angelo D.F.S., Rabello V.B.S., Maciel M.A.S., Atanázio S.S.L.A., Costa M.C.L., Silva S.R., Silva S.R., Almeida-Paes R., Bernardes-Engemann A.R., Zancopé-Oliveira R.M. (2023). *Sporothrix brasiliensis* infecting cats in northeastern Brazil: New emerging areas in Paraíba state. Cienc. Rural.

[7] de Oliveira Bento A., de Sena Costa A.S., Lima S.L., Alves M.M., Melo A.S.A., Rodrigues A.M., Silva-Rocha W.P., Milan E.P., Chaves G.M. (2021). The spread of cat-transmitted sporotrichosis due to *Sporothrix brasiliensis* in brazil towards the northeast region. PLoS Negl. Trop. Dis..

[8] de Oliveira P.R.F., de Carvalho J.A., Costa T.R., Silva B.P.E., da Silva G.G., Rodrigues A.M., Mota R.A. (2024). Emerging cases of cat-transmitted sporotrichosis driven by *Sporothrix brasiliensis* in northeast brazil. Mycopathologia.

[9] de Carvalho J.A., Hagen F., Fisher M.C., de Camargo Z.P., Rodrigues A.M. (2020). Genome-wide mapping using new AFLP markers to explore intraspecific variation among pathogenic *Sporothrix* species. PLoS Negl. Trop. Dis..

[10] Roberto T.N., de Carvalho J.A., Beale M.A., Hagen F., Fisher M.C., Hahn R.C., de Camargo Z.P., Rodrigues A.M. (2021). Trends in the molecular epidemiology and population genetics of emerging *Sporothrix* species. Stud. Mycol..

[11] Gonçalves S.S., da Cruz Bahiense Rocha I., Redigueri B.C., de Carvalho J.A., Maifrede S.B., Kruschewsky W.L.L., Falqueto A., Rodrigues A.M. (2023). Human and feline sporotrichosis in a reference center of Southeastern Brazil: Genetic differentiation, diversity, and antifungal susceptibility of *Sporothrix* species. J. Fungi.

[12] Aguiar B.A., Borges I.L., Silva B.W.L., Rodrigues F.R.N., Gonçalves L.D., Casseb A.R., Brito J.S., Pinheiro A.Q., Rocha M.F.G., Viana D.A. (2023). First case report of feline sporotrichosis caused by *Sporothrix brasiliensis* in the state of Ceará–Brazil. Med. Mycol. Case Rep..

[13] Damasceno L.S., de Almeida Junior A.M.B., Farias L.A.B.G., de Mesquita J.R.L., Almeida M.A., de Almeida Paes R., Zancopé-Oliveira R.M. (2023). Esporotricose: Uma Micose em Expansão no Ceará. Braz. J. Infect. Dis..

[14] Instituto Brasileiro de Geografia e Estatística (2023). Censo Demográfico 2022: Indicadores de Saneamento e Renda. https://www.ibge.gov.br.

[15] Instituto de Pesquisa e Estratégia Econômica do Ceará (2024). Informe de Pobreza e Desigualdade no Ceará. https://www.ipece.ce.gov.br.

[16] Observatório das Metrópoles (2026). Boletim Desigualdade Nas Metrópoles: N° 17—Edição Especial—Dados Anuais (2012–2025). https://www.observatoriodasmetropoles.net.br/wp-content/uploads/2026/06/boletim_n17_jun26.pdf.

[17] Scuarcialupi L.N., Pereira F.C., Baquero O.S. (2021). Feline sporotrichosis: Social vulnerability and prioritization of geographic areas in Guarulhos, SP, Brazil. Braz. J. Vet. Res. Anim. Sci..

[18] Scuarcialupi L.N., Moraes G.C., Pereira F.C., Alexandre Y.S., Baquero O.S. (2025). Epidemiological surveillance of neglected tropical diseases in silent areas: The case of zoonotic sporotrichosis. Cienc. Saude Colet..

[19] Hyde R.M., Down P.M., Bradley A.J., Breen J.E., Hudson C., Leach K.A., Green M.J. (2020). Automated prediction of mastitis infection patterns in dairy herds using machine learning. Sci. Rep..

[20] Oehm A.W., Zablotski Y., Campe A., Hoedemaker M., Strube C., Springer A., Jordan D., Knubben-Schweizer G. (2023). Random forest classification as a tool in epidemiological modelling: Identification of farm-specific characteristics relevant for the occurrence of *Fasciola hepatica* on German dairy farms. PLoS ONE.

[21] Hosmer D.W., Lemeshow S., Sturdivant R.X. (2013). Applied Logistic Regression.

[22] Breiman L. (2001). Random forests. Mach. Learn..

[23] Robinson W.S. (2009). Ecological correlations and the behavior of individuals. Int. J. Epidemiol..

[24] Diez Roux A.V. (2002). A glossary for multilevel analysis. J. Epidemiol. Community Health.

[25] Prefeitura Municipal de Fortaleza, n.d. Fortaleza em Bairros. https://fortalezaembairros.fortaleza.ce.gov.br/.

[26] Secretaria Municipal da Saúde Sistema de Monitoramento Diário de Agravos (SIMDA).

[27] Gibbons C.L., Mangen M.-J.J., Plass D., Havelaar A.H., Brooke R.J., Kramarz P., Peterson K.L., Stuurman A.L., Cassini A., Fèvre E.M. (2014). Measuring underreporting and under-ascertainment in infectious disease datasets: A comparison of methods. BMC Public Health.

[28] Wilson E.B. (1927). Probable inference, the law of succession, and statistical inference. J. Am. Stat. Assoc..

[29] Brown L.D., Cai T.T., DasGupta A. (2001). Interval estimation for a binomial proportion. Stat. Sci..

[30] Sidrim J.J.C., Rocha M.F.G. (2004). Micologia Médica À Luz de Autores Contemporâneos.

[31] Marimon R., Cano J., Gené J., Sutton D.A., Kawasaki M., Guarro J. (2007). *Sporothrix brasiliensis*, *S. globosa*, and *S. mexicana*, three new *Sporothrix* species of clinical interest. J. Clin. Microbiol..

[32] Graser A., Peterson G.N. (2018). QGIS Map Design.

[33] Zar J.H. (2010). Biostatistical Analysis.

[34] Benjamini Y., Yekutieli D. (2001). The control of the false discovery rate in multiple testing under dependency. Ann. Stat..

[35] Kuhn M., Johnson K. (2013). Applied Predictive Modeling.

[36] Hastie T., Tibshirani R., Friedman J. (2009). The Elements of Statistical Learning: Data Mining, Inference, and Prediction.

[37] Jolliffe I.T. (2002). Principal Component Analysis.

[38] Varma S., Simon R. (2006). Bias in error estimation when using cross-validation for model selection. BMC Bioinform..

[39] Cawley G.C., Talbot N.L.C. (2010). On over-fitting in model selection and subsequent selection bias in performance evaluation. J. Mach. Learn. Res..

[40] Gneiting T., Raftery A.E. (2007). Strictly proper scoring rules, prediction, and estimation. J. Am. Stat. Assoc..

[41] Scikit-Learn Developers Common Pitfalls and Recommended Practices. In *Scikit-Learn Documentation*. https://scikit-learn.org/stable/common_pitfalls.html.

[42] Zou H., Hastie T. (2005). Regularization and variable selection via the elastic net. J. R. Stat. Soc. Ser. B Stat. Methodol..

[43] Fawcett T. (2006). An introduction to ROC analysis. Pattern Recognit. Lett..

[44] Saito T., Rehmsmeier M. (2015). The precision-recall plot is more informative than the roc plot when evaluating binary classifiers on imbalanced datasets. PLoS ONE.

[45] Lundberg S.M., Lee S.-I. (2017). A unified approach to interpreting model predictions. Proceedings of Advances in Neural Information Processing Systems.

[46] Harris C.R., Millman K.J., van der Walt S.J., Gommers R., Virtanen P., Cournapeau D., Wieser E., Taylor J., Berg S., Smith N.J. (2020). Array programming with NumPy. Nature.

[47] McKinney W., van der Walt S., Millman J. (2010). Data structures for statistical computing in Python. Proceedings of the 9th Python in Science Conference.

[48] Pedregosa F., Varoquaux G., Gramfort A., Michel V., Thirion B., Grisel O. (2011). Scikit-learn: Machine learning in Python. J. Mach. Learn. Res..

[49] Rossow J.A., Queiroz-Telles F., Caceres D.H., Beer K.D., Jackson B.R., Pereira J.G., Gremião I.D.F., Pereira S.A. (2020). A one health approach to combatting *Sporothrix brasiliensis*: Narrative review of an emerging zoonotic fungal pathogen in South America. J. Fungi.

[50] Xavier M.O., Poester V.R., Trápaga M.R., Stevens D.A. (2023). *Sporothrix brasiliensis*: Epidemiology, therapy, and recent developments. J. Fungi.

[51] Schober P., Boer C., Schwarte L.A. (2018). Correlation coefficients: Appropriate use and interpretation. Anesth. Analg..

[52] Wasserstein R.L., Lazar N.A. (2016). The ASA statement on *p*-values: Context, process, and purpose. Am. Stat..

[53] Boechat J.S., Oliveira M.M.E., Almeida-Paes R., Gremião I.D.F., Machado A.C.S., Oliveira R.V.C., Figueiredo A.B.F., Rabello V.B.S., Silva K.B.L., Zancopé-Oliveira R.M. (2018). Feline sporotrichosis: Associations between clinical-epidemiological profiles and phenotypic-genotypic characteristics of the etiological agents in the Rio de Janeiro epizootic area. Mem. Inst. Oswaldo Cruz.

[54] Prohaska-Batista F., Cavassin F.B., Queiroz-Telles F. (2025). From neglected to notable: A growing public health challenge driven by hospitalization for sporotrichosis in Pernambuco, Northeast Brazil (2016–2024). PLoS Negl. Trop. Dis..

[55] Bastos F.A.D.G., Farias M.R., Gremião I.D.F., Cavassin F.B., Monti F.S., Vilas Boas R.R., Lustosa B.P.R., Razzolini E., Vicente V.A., Queiroz-Telles F. (2025). Cat-transmitted sporotrichosis by *Sporothrix brasiliensis*: Focus on its potential transmission routes and epidemiological profile. Med. Mycol..

[56] Ministério da Saúde Esporotricose Humana [Human Sporotrichosis]. https://www.gov.br/saude/pt-br/assuntos/saude-de-a-a-z/e/esporotricose-humana.

[57] Nakasu C.C.T., Waller S.B., Ripoll M.K., Ferreira M.R.A., Conceição F.R., Gomes A.D.R., da Gama Osório L., de Faria R.O., Cleff M.B. (2021). Feline sporotrichosis: A case series of itraconazole-resistant *Sporothrix brasiliensis* infection. Braz. J. Microbiol..

