# Supplementary material for: Emerging Zoonotic Sporotrichosis in Fortaleza, Brazil: Spatiotemporal Distribution, Exploratory Machine Learning Risk Modeling, and One Health Implications of Sporothrix brasiliensis Detection
Source: Animals (Basel). 2026 Nov 5;16(21):3498. doi: 10.3390/ani16213498 (PMC13761459; doi:10.3390/ani16213498)
Supplement: Supplementary file 1 [file animals-16-03498-s001.zip › animals-4591853-supplementary.pdf]

Supplementary Materials

Table S1. Official neighborhoods of Fortaleza merged into a single row in the database (CSV), with the row identification and the criterion indicating the merge**.**

CSV row (ID)	Official neighborhoods merged	Evidence
CONJUNTO CEARÁ	Conjunto Ceará I + Conjunto Ceará II	Single row with no I/II suffix
PRAIA DO FUTURO	Praia do Futuro I + Praia do Futuro II	Single row with no I/II suffix
QUINTINO CUNHA (OLAVO OLIVEIRA)	Quintino Cunha + Olavo Oliveira	Second name written in parentheses
MONDUBIM	Mondubim + Novo Mondubim	Single row, with no distinction
ANCURI	Ancuri + Santa Maria	Single row, with no distinction

Table S2. Temporal and geographic distribution of isolates tested by species-specific PCR relative to culture-confirmed feline cases (n = 120), Fortaleza, January 2022 to March 2026.

Year		Isolates tested by PCR (n)	Tested (%)	Neighborhoods of tested isolates (n)
**2022**	2	2	100.0	Jangurussu (2)
**2023**	0	0	—	—
**2024**	1	0	0.0	— (neighborhood not reported)
**2025**	61	16	26.2	Barra do Ceará (3), Jangurussu (3), Vila Velha (2), Messejana (2), Planalto Ayrton Senna (1), Fátima (1), Conjunto Palmeiras (1), Bom Jardim (1), Aldeota (1), Dionísio Torres (1)
**2026 (Jan–Mar)**	56	19	33.9	Jangurussu (4), Vila Velha (4), Messejana (3), Jardim Guanabara (1), Jardim Iracema (1), Canindezinho (1), Henrique Jorge (1), Rodolfo Teófilo (1), Montese (1), José Bonifácio (1), Ancuri (1)
**Total**	**120**	**37**	**30.8**	18 neighborhoods

Note: The seven confirmed cases without neighborhood information are included in the annual totals of confirmed cases.

The following supporting information is available: Table S3: variables used in the neighborhood-level and animal-level models (definition, coding, and percentage of missing data); Figure S1: flow of animals and neighborhoods in each analysis.:

Table S3. Variables used in the neighborhood-level and animal-level models: definition, coding, and percentage of missing data. Missing data were kept as a "not reported" category and were not imputed.

Level	Variable	Definition	Coding	Missing (%)
**Neighborhood**	HDI-Income	Income component of the HDI	0–1	0
**Neighborhood**	Population density	Inhabitants/km²	Continuous	0
**Neighborhood**	Precarious settlements	Precarious settlement level	Ordinal, 0–5	0
**Neighborhood**	Vegetation cover	m² per inhabitant	Continuous	0
**Neighborhood**	Animal / human health units	Number of units in the neighborhood	Count	0
**Neighborhood**	Latitude, longitude, residents per lot, access to public	Geographic latitude/longitude of the neighborhood	Continuous	0
**Neighborhood**	Residents per lot	Average number of residents per habitational unit	Continuous	0
**Neighborhood**	Access to public squares	Percentage of residents in a neighborhood who live within 300 meters of a public square.	Continuous	0
**Animal**	Sex	Questionnaire	Male / female / not reported	9.2
**Animal**	Neutering status	Questionnaire	Neutered / unneutered / not reported	13.8
**Animal**	Kitten	Age ≤6 months	Kitten / adult / not reported	11.3
**Animal**	Habit	Usual rearing system	Strictly indoor / semi-domiciled / free-roaming / not reported	4.2
**Animal**	Exposure	Reported contact with animals with suspected or confirmed sporotrichosis (street access not included)	Yes / no / not reported	8.4
**Animal**	Suspected household pets	Other animals with suspected sporotrichosis in the same household (also classified as exposure)	Yes / no / not reported	36.8
**Animal**	Animal hoarding	≥5 animals in the household	Yes / no / not reported	25.5

**Animal**	Ulcer distribution	Localized (≤2 lesions at the same site) or disseminated (>2 lesions at distinct sites)	Localized / disseminated / not reported	32.2

Note: Percentages of missing data for animal-level variables refer to the 239 cats investigated.

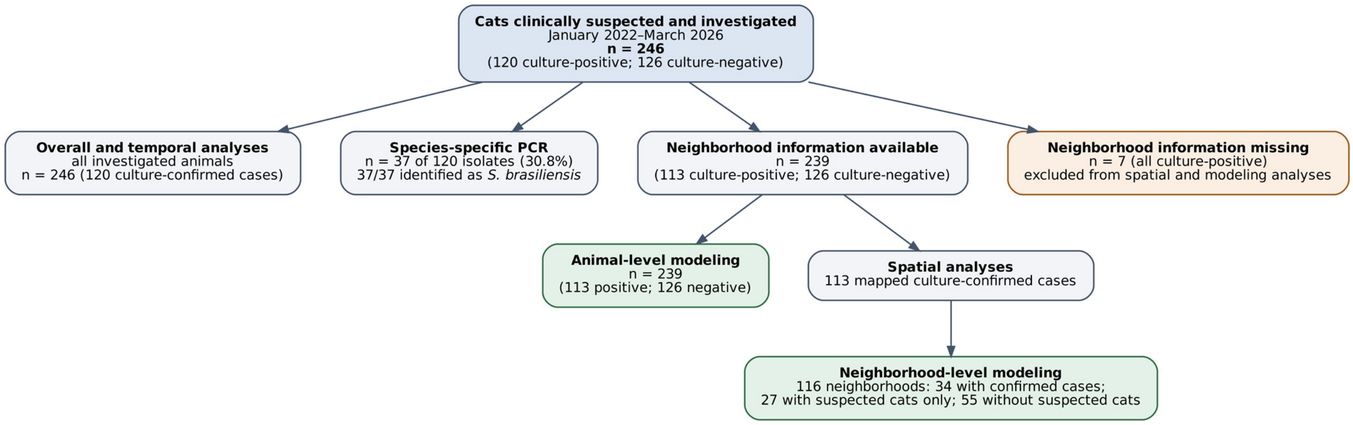

Figure S1. Flow of animals and neighborhoods in each analysis. Of the 246 cats investigated, 120 were culture-positive; of these, 37 (30.8%) underwent species-specific PCR. Seven confirmed cats without neighborhood information were included only in the overall and temporal analyses. The 239 cats with neighborhood information (113 positive and 126 negative) comprised the animal-level model, and the 113 mapped confirmed cases comprised the spatial analyses. The neighborhood-level model used 116 neighborhoods (34 positive).

Table S4. Classification of the 126 culture-negative cats.

Category	Specification	n	% of 126
Re-sampling not obtained	Contaminated culture	9	7.1
Inadequate specimen	Insufficient material	0	0
	Sample decomposition	3	2.4
	Delayed transport	2	1.6
	Adherent slides	1	0.8
	Absence of slide or swab	0	0
	**Subtotal**	15	11.9
Alternative infectious diagnosis	*Histoplasma* spp.	3	2.4
	Microsporum canis	8	6.3
	Trichophyton spp.	1	0.8
	Cryptococcus spp.	3	2.4
	Leishmania spp.	1	0.8
	Candida spp.	2	1.6
	Malassezia spp.	1	0.8
	**Subtotal**	19	15.1

Alternative neoplastic diagnosis	Squamous cell carcinoma	7	5.6
Etiology undetermined	—	85	67.5
**Total**		**126**	**100%**

Note: Categories are mutually exclusive. Each cat was assigned according to its final classification after re-sampling, which was requested for inadequate specimens and contaminated cultures whenever possible. Cats culture-confirmed after re-sampling are not included.

Table S5. Investigated (suspected) cats, culture-confirmed cats, human sporotrichosis notifications, and population by neighborhood in Fortaleza (n = 116), January 2022 to March 2026.

Neighborhood	Population	Investigated (suspected) cats, n	Confirmed cats, n	Human notifications, n	Confirmed cats per 100,000 inhabitants	Human notifications per 100,000 inhabitants
**Vila Velha**	52,881	32	18	18	34.0	34.0
**Jangurussu**	70,651	22	17	6	24.1	8.5
**Messejana**	43,205	16	9	2	20.8	4.6
**Mondubim**	62,440	14	8	2	12.8	3.2
**Barra do Ceará**	63,477	12	7	2	11.0	3.2
**Conjunto Palmeiras**	28,223	10	7	9	24.8	31.9
**Paupina**	26,446	11	6	3	22.7	11.3
**Coaçu**	7,132	6	5	1	70.1	14.0
**Álvaro Weyne**	21,809	6	3	1	13.8	4.6
**Aldeota**	42,580	3	3	1	7.0	2.3
**Jardim Guanabara**	12,299	16	2	2	16.3	16.3
**Passaré**	54,870	6	2	0	3.6	0.0
**Rachel de Queiroz**	24,850	6	2	0	8.0	0.0
**Ancuri**	7,559	3	2	2	26.5	26.5
**Jardim Iracema**	19,005	2	2	4	10.5	21.0
**Montese**	21,794	2	2	0	9.2	0.0
**Serrinha**	27,889	4	1	0	3.6	0.0
**Itaperi**	22,213	3	1	1	4.5	4.5
**Curió**	6,517	3	1	0	15.3	0.0
**Jacarecanga**	13,063	3	1	0	7.7	0.0
**Sapiranga/Coité**	29,215	3	1	0	3.4	0.0
**Antônio Bezerra**	23,186	2	1	0	4.3	0.0
**Bom Jardim**	33,720	2	1	0	3.0	0.0

**Fátima**	23,114	2	1	0	4.3	0.0
**José Bonifácio**	8,539	2	1	0	11.7	0.0
**São Bento**	12,247	1	1	2	8.2	16.3
**Rodolfo Teófilo**	16,067	1	1	1	6.2	6.2
**Autran Nunes**	18,710	1	1	0	5.3	0.0
**Bonsucesso**	35,766	1	1	0	2.8	0.0
**Canindezinho**	37,113	1	1	0	2.7	0.0
**Dias Macedo**	11,855	1	1	0	8.4	0.0
**Dionísio Torres**	13,875	1	1	0	7.2	0.0
**Henrique Jorge**	24,434	1	1	0	4.1	0.0
**Planalto Ayrton Senna**	37,890	1	1	0	2.6	0.0
**Maraponga**	12,482	4	0	0	0.0	0.0
**Granja Lisboa**	63,420	3	0	1	0.0	1.6
**Parque Dois Irmãos**	32,065	3	0	0	0.0	0.0
**Vicente Pinzon**	46,151	2	0	1	0.0	2.2
**Aracapé**	18,943	2	0	0	0.0	0.0
**Cajazeiras**	13,852	2	0	0	0.0	0.0
**Jardim Cearense**	10,898	2	0	0	0.0	0.0
**Parque São José**	7,645	2	0	0	0.0	0.0
**Barroso**	31,467	1	0	2	0.0	6.4
**Centro**	24,096	1	0	2	0.0	8.3
**Aerolândia**	9,368	1	0	0	0.0	0.0
**Bela Vista**	14,648	1	0	0	0.0	0.0
**Cidade dos Funcionários**	15,486	1	0	0	0.0	0.0
**Cocó**	23,005	1	0	0	0.0	0.0
**Conjunto Ceará**	17,594	1	0	0	0.0	0.0
**Edson Queiroz**	24,850	1	0	0	0.0	0.0
**Genibau**	35,435	1	0	0	0.0	0.0
**Granja Portugal**	36,619	1	0	0	0.0	0.0
**Guajeru**	6,432	1	0	0	0.0	0.0
**José Walter**	53,455	1	0	0	0.0	0.0

**Monte Castelo**	11,267	1	0	0	0.0	0.0
**Panamericano**	8,580	1	0	0	0.0	0.0
**Parangaba**	27,988	1	0	0	0.0	0.0
**Pici**	38,377	1	0	0	0.0	0.0
**Presidente Vargas**	10,906	1	0	0	0.0	0.0
**Vila Peri**	17,550	1	0	0	0.0	0.0
**Vila União**	13,275	1	0	0	0.0	0.0
**Carlito Pamplona**	23,673	0	0	1	0.0	4.2
**Floresta**	24,363	0	0	1	0.0	4.1
**Joaquim Távora**	20,749	0	0	1	0.0	4.8
**João XXIII**	16,475	0	0	1	0.0	6.1
**Praia de Iracema**	3,033	0	0	1	0.0	33.0
**Aeroporto**	8,995	0	0	0	0.0	0.0
**Alto da Balanca**	10,407	0	0	0	0.0	0.0
**Amadeu Furtado**	9,681	0	0	0	0.0	0.0
**Benfica**	12,511	0	0	0	0.0	0.0
**Boa Vista/Castelão**	12,328	0	0	0	0.0	0.0
**Bom Futuro**	5,260	0	0	0	0.0	0.0
**Cais do Porto**	22,749	0	0	0	0.0	0.0
**Cambeba**	8,775	0	0	0	0.0	0.0
**Cidade 2000**	7,835	0	0	0	0.0	0.0
**Conjunto Esperança**	12,729	0	0	0	0.0	0.0
**Couto Fernandes**	4,645	0	0	0	0.0	0.0
**Cristo Redentor**	24,580	0	0	0	0.0	0.0
**Damas**	10,148	0	0	0	0.0	0.0
**De Lourdes**	2,310	0	0	0	0.0	0.0
**Demócrito Rocha**	10,460	0	0	0	0.0	0.0
**Dom Lustosa**	12,370	0	0	0	0.0	0.0
**Ellery**	6,784	0	0	0	0.0	0.0
**Farias Brito**	10,384	0	0	0	0.0	0.0
**Guararapes**	7,642	0	0	0	0.0	0.0

**Itaoca**	10,544	0	0	0	0.0	0.0
**Jardim América**	10,286	0	0	0	0.0	0.0
**Jardim das Oliveiras**	26,351	0	0	0	0.0	0.0
**Joquei Clube**	20,249	0	0	0	0.0	0.0
**José de Alencar**	14,651	0	0	0	0.0	0.0
**Lagoa Redonda**	30,410	0	0	0	0.0	0.0
**Luciano Cavalcante**	21,779	0	0	0	0.0	0.0
**Manuel Dias Branco**	7,762	0	0	0	0.0	0.0
**Meireles**	38,374	0	0	0	0.0	0.0
**Moura Brasil**	3,029	0	0	0	0.0	0.0
**Mucuripe**	13,775	0	0	0	0.0	0.0
**Padre Andrade**	10,860	0	0	0	0.0	0.0
**Papicu**	20,728	0	0	0	0.0	0.0
**Parque Araxá**	6,100	0	0	0	0.0	0.0
**Parque Iracema**	13,669	0	0	0	0.0	0.0
**Parque Manibura**	5,938	0	0	0	0.0	0.0
**Parque Santa Rosa**	11,792	0	0	0	0.0	0.0
**Parquelândia**	12,842	0	0	0	0.0	0.0
**Parreão**	9,132	0	0	0	0.0	0.0
**Pedras**	17,101	0	0	0	0.0	0.0
**Pirambu**	14,176	0	0	0	0.0	0.0
**Praia do Futuro**	4,329	0	0	0	0.0	0.0
**Presidente Kennedy**	22,685	0	0	0	0.0	0.0
**Quintino Cunha**	30,324	0	0	0	0.0	0.0
**Sabiaguaba**	3,485	0	0	0	0.0	0.0
**Salinas**	4,837	0	0	0	0.0	0.0
**Siqueira**	45,494	0	0	0	0.0	0.0
**São Gerardo**	15,761	0	0	0	0.0	0.0
**São João do Tauape**	23,426	0	0	0	0.0	0.0
**Varjota**	6,703	0	0	0	0.0	0.0
**Vila Manoel Sátiro**	16,073	0	0	0	0.0	0.0

**Total**	**2,362,039**	**239**	**113**	**68**	**4.8**	**2.9**

Note: Investigated cats include confirmed cases. Seven confirmed cats lacking neighborhood information are not included. Rates were calculated as (cases ÷ population) × 100,000 and are unstable in neighborhoods with small populations (e.g., Praia de Iracema, 3,033 inhabitants, 1 notification, 33.0 per 100,000). The number of confirmed cats depends on the number of cats investigated in each neighborhood, which varies with surveillance intensity. Sources: SIMDA (human notifications, 2024–2026) and Demographic Census (population). The 116 neighborhoods result from the merging of adjacent official neighborhoods.

Table S6. Characteristics of the cats investigated according to culture result, Fortaleza, January 2022 to March 2026 (n = 246).

Variable	Positive (n = 120), n (%)	Negative (n = 126), n (%)
**Sex**		
Male	60 (50.0)	54 (42.9)
Female	51 (42.5)	58 (46.0)
NR	9 (7.5)	14 (11.1)
**Neutering status**		
Neutered	44 (36.7)	60 (47.6)
Unneutered	55 (45.8)	48 (38.1)
NR	21 (17.5)	18 (14.3)
**Sex and neutering status**		
Unneutered male	36 (30.0)	27 (21.4)
Neutered male	18 (15.0)	24 (19.0)
Unneutered female	16 (13.3)	21 (16.7)
Neutered female	26 (21.7)	36 (28.6)
Neutering NR (known sex)	15 (12.5)	4 (3.2)
Sex NR	9 (7.5)	14 (11.1)
**Age**		
Adult	105 (87.5)	103 (81.7)
Kitten (≤6 months)	5 (4.2)	3 (2.4)
NR	10 (8.3)	20 (15.9)
**Lesion distribution**		
Localized	35 (29.2)	37 (29.4)
Disseminated	54 (45.0)	42 (33.3)
NR*	31 (25.8)	47 (37.3)
**Habit**		

Strictly indoor	43 (35.8)	65 (51.6)
Semi-domiciled+free-roaming	67 (55.8)	58 (46.0)
NR	10 (8.3)	3 (2.4)
**Animal hoarding (≥5 animals in the household)**		
Yes	45 (37.5)	56 (44.4)
No	34 (28.3)	46 (36.5)
NR	41 (34.2)	24 (19.0)
**Exposure (contact with suspected or infected animals)**		
Exposed	69 (57.5)	79 (62.7)
Not exposed	32 (26.7)	42 (33.3)
NR	19 (15.8)	5 (4.0)
**Suspected animals in the household**		
Yes	29 (24.2)	25 (19.8)
No	48 (40.0)	52 (41.3)
NR	43 (35.8)	49 (38.9)

*Includes the asymptomatic cat, classified as “not reported” in the models. *Note:* Percentages are calculated using the total of each group as denominator and include missing data (NR = not reported).

Table S7A. Cats investigated, culture-confirmed cases, and surveillance strategy by period, Fortaleza, January 2022 to March 2026.

Period	Cats investigated (n)	Culture-confirmed (n)	Confirmation, % (95% Wilson CI)	Surveillance
2022	2	2	100.0 (34.2–100.0)	Passive
2023	0	0	—	Passive
2024	1	1	100.0 (20.7–100.0)	Passive
2025	101	61	60.4 (50.6–69.4)	Passive + localized active
2026	142	56	39.4 (31.8–47.7)	Passive + systematic active
**Total**	**246**	**120**	**48.8 (42.6–55.0)**	

Note: The capture route (passive or active) was not recorded for each animal; the last column describes the surveillance strategy in place during each period.

Table S7B. Selected hyperparameters in each outer fold of nested cross-validation

A. Logistic Regression

Outer fold	Solver	Penalty	C	class_weight	max_iter	Outer fold accuracy (%)
**Neighborhood, 10 predictors (n = 61)**						
1	liblinear	L2	10	balanced	500	41.67
2	saga	L2	10	balanced	1000	45.45
3	liblinear	L1	10	balanced	500	27.27
4	liblinear	L2	10	balanced	500	30.77
5	liblinear	L2	10	none	500	28.57
**Neighborhood, 8 predictors, without health units (n = 61)**						
1	liblinear	L1	10	balanced	1000	25.00
2	saga	L2	10	balanced	500	36.36
3	liblinear	L1	10	balanced	1000	45.45
4	saga	L2	10	balanced	1000	53.85
5	liblinear	L1	10	none	500	28.57
**Individual, all cats with neighborhood (n = 239)**						
1	liblinear	L1	10	balanced	500	57.35
2	saga	L1	10	balanced	500	44.44
3	liblinear	L1	10	balanced	1000	50.00
4	liblinear	L1	10	balanced	500	42.37
5	saga	L2	10	none	500	45.16
**Individual, complete cases (n = 120)**						
1	liblinear	L1	10	balanced	1000	46.15
2	liblinear	L1	10	balanced	1000	33.33
3	saga	L1	10	none	500	38.46
4	liblinear	L1	10	balanced	500	33.33
5	liblinear	L1	10	balanced	500	39.02

$C = 1/\lambda$. In all 20 fits, the selected value was $C = 10$, the upper regularization limit of the grid ($\lambda = 0.1$). Elastic-net penalty and l1_ratio were part of the grid but were not selected in any fold.

B. Random Forest

Outer fold	Criterion	max_depth	max_features	min_samples_leaf	class_weight	Outer fold accuracy (%)
**Neighborhood, 10 predictors (n = 61)**						
1	entropy	3	sqrt	3	balanced	16.67
2	entropy	3	sqrt	2	balanced	81.82
3	gini	3	sqrt	2	balanced	54.55
4	gini	10	sqrt	2	balanced	69.23
5	gini	3	sqrt	2	balanced	57.14
**Neighborhood, 8 predictors, without health units (n = 61)**						
1	entropy	3	log2	3	balanced	16.67
2	gini	3	log2	2	balanced	63.64
3	entropy	3	sqrt	3	balanced	72.73
4	gini	5	sqrt	3	balanced	69.23
5	gini	3	sqrt	3	balanced	57.14
**Individual, all cats with neighborhood (n = 239)**						
1	gini	3	sqrt	3	balanced	55.88
2	gini	3	log2	3	none	52.38
3	gini	5	log2	3	balanced	44.44
4	entropy	3	log2	3	balanced	47.46
5	entropy	3	sqrt	2	balanced	41.94
**Individual, complete cases (n = 120)**						
1	gini	5	sqrt	3	balanced	53.85
2	entropy	3	sqrt	2	balanced	46.67
3	gini	3	sqrt	3	none	35.90
4	entropy	3	sqrt	2	balanced	41.67
5	entropy	3	sqrt	2	balanced	53.66

All models: n_estimators = 100 (scikit-learn default) and random_state = 12345.

General Note. Hyperparameters were selected in the inner loop (5 folds, StratifiedGroupKFold, shuffled, seed 12345), minimizing log loss, using only the training data from the outer fold. Groups corresponded to the 12 administrative regions (neighborhood level) and the 61 neighborhoods (individual level). Accuracy was evaluated on the outer test fold (decision threshold 0.5) with the model refitted on the selected hyperparameters; test folds are small (neighborhood level: 11–14 observations), which explains the high variation in accuracy across

folds. Search grids: logistic regression, 200 combinations (liblinear: l1/l2; lbfgs: l2/none; saga: elastic-net with l1_ratio 0.25/0.5/0.75, l1, l2, and none; C = 1/λ with $\lambda = 10^{-5}$, 10^{-4}, 10^{-3}, 10^{-2}, 0.1; max_iter 500 or 1000; class_weight balanced or none). Random forest, 96 combinations (criterion gini/entropy/log_loss; max_depth 3/5/10/15; min_samples_leaf 2/3; max_features sqrt/log2; class_weight balanced or none).

Table S8. Exploratory Spearman rank correlations, 95% cluster-bootstrap confidence intervals, and Benjamini–Yekutieli multiplicity-adjusted p-values between socio-environmental and individual predictors and feline sporotrichosis detection outcomes in Fortaleza, Brazil**.**

Analysis Level	Variable	N	Groups	Spearman Rho	95% CI Lower	95% CI Upper	Conventional p-value	BY Adjusted p-value	Valid Bootstraps (B)	Invalid Fraction	BY Origin / Status
Neighborhood (Territorial)	HUMAN_HEALTH_UNITS	61	12	0.120	-0.178	0.366	0.357	1.000	5000	0.0%	BY verified; stored value
Neighborhood (Territorial)	PRECARIOUS_SETTLEMENT_LEVEL	61	12	0.183	-0.064	0.418	0.159	1.000	5000	0.0%	BY verified; stored value
Neighborhood (Territorial)	ANIMAL_HEALTH_UNITS	61	12	0.114	-0.113	0.271	0.383	1.000	4989	0.2%	BY verified; stored value
Neighborhood (Territorial)	RESIDENTS_PER_LOT	61	12	0.124	-0.190	0.386	0.342	1.000	5000	0.0%	BY verified; stored value
Neighborhood (Territorial)	VEGETATION_COVERAGE	61	12	-0.025	-0.283	0.176	0.846	1.000	5000	0.0%	BY verified; stored value
Neighborhood (Territorial)	ACCESS_TO_SQUARES	61	12	-0.042	-0.343	0.271	0.747	1.000	5000	0.0%	BY verified; stored value
Neighborhood (Territorial)	POPULATION_DENSITY	61	12	0.030	-0.188	0.298	0.819	1.000	5000	0.0%	BY verified; stored value
Neighborhood (Territorial)	LONGITUDE	61	12	0.131	-0.161	0.337	0.313	1.000	5000	0.0%	BY verified; stored value
Neighborhood (Territorial)	LATITUDE	61	12	0.001	-0.384	0.366	0.994	1.000	5000	0.0%	BY verified; stored value
Neighborhood (Territorial)	HDI-INCOME	61	12	-0.083	-0.301	0.141	0.527	1.000	5000	0.0%	BY verified; stored value
Individual (Expanded Dataset)	SEX	217	61	0.080	-0.051	0.224	0.242	1.000	5000	0.0%	BY verified; stored value

Individual (Expanded Dataset)	CASTRATED	206	60	-0.106	-0.300	0.064	0.128	1.000	5000	0.0%	BY verified; stored value
Individual (Expanded Dataset)	KITTEN	212	61	0.050	-0.084	0.180	0.473	1.000	5000	0.0%	BY verified; stored value
Individual (Expanded Dataset)	HABIT	152	48	-0.135	-0.328	0.059	0.096	1.000	5000	0.0%	BY verified; stored value
Individual (Expanded Dataset)	SUSPECTED HOUSEHOLD PETS	151	53	0.056	-0.122	0.225	0.493	1.000	5000	0.0%	BY verified; stored value
Individual (Expanded Dataset)	EXPOSURE	219	60	0.022	-0.124	0.157	0.750	1.000	5000	0.0%	BY verified; stored value
Individual (Expanded Dataset)	ANIMAL HOARDING	178	51	0.030	-0.140	0.201	0.693	1.000	5000	0.0%	BY verified; stored value
Individual (Expanded Dataset)	ULCER DISTRIBUTION	162	56	0.096	-0.074	0.259	0.224	1.000	5000	0.0%	BY verified; stored value
Individual (Complete Cases)	SEX	120	47	0.162	-0.026	0.342	0.077	1.000	5000	0.0%	BY calculated in this release with rounded p
Individual (Complete Cases)	CASTRATED	120	47	-0.105	-0.371	0.138	0.254	1.000	5000	0.0%	BY calculated in this release with rounded p
Individual (Complete Cases)	KITTEN	120	47	0.105	-0.006	0.237	0.252	1.000	4926	1.5%	BY calculated in this release with rounded p
Individual (Complete Cases)	HABIT	78	35	0.006	-0.226	0.284	0.956	1.000	5000	0.0%	BY calculated in this release with rounded p
Individual (Complete Cases)	SUSPECTED HOUSEHOLD PETS	120	47	0.051	-0.133	0.227	0.581	1.000	5000	0.0%	BY calculated in this release with rounded p
Individual (Complete Cases)	EXPOSURE	120	47	-0.005	-0.189	0.140	0.961	1.000	5000	0.0%	BY calculated in this release with rounded p
Individual (Complete Cases)	ANIMAL HOARDING	120	47	0.096	-0.093	0.293	0.287	1.000	5000	0.0%	BY calculated in this release with rounded p

Individual (Complete Cases)	ULCER DISTRIBUTION	120	47	0.022	-0.159	0.210	0.809	1.000	5000	0.0%	BY calculated in this release with rounded p

Note: Percentile bootstrap confidence intervals (B = 5,000, seed = 12345) were estimated by resampling administrative regions (neighborhood-level) and neighborhoods (individual-level). Conventional p-values are unadjusted for grouping. Benjamini–Yekutieli (BY) adjustments were performed separately for each family of tests (10 territorial, 8 expanded individual, and 8 complete-case individual contrasts).

Table S9A. SHAP Feature Stability – Neighborhood Level (Logistic Regression).

Variable	Median position	Best position	Top k folds	N folds	n_folds	Top k frequency
**LONGITUDE**	2.0	1.0	2.0	5	5	1.0
**PRECARIOUS_SETTLEMENT_LEVEL**	2.0	1.0	5.0	3	5	0.6
**VEGETETIONS_COVERAGE**	4.0	3.0	6.0	2	5	0.4
**POPULATION_DENSITY**	5.0	4.0	6.0	0	5	0.0
**ACCES_TO _SQUARES**	6.0	3.0	7.0	1	5	0.2
**RESIDENTS_PER_LOT**	7.0	1.0	8.0	2	5	0.4
**LATITUDE**	7.0	2.0	8.0	1	5	0.2
**HDI-INCOME**	7.0	3.0	8.0	1	5	0.2

Table S9B. SHAP Feature Stability – Neighborhood Level (Radom Forest).

Variable	Median position	Best position	Top k folds	N folds	n_folds	Top k frequency
**LATITUDE**	2.0	1.0	6.0	4	5	2.0
**RESIDENTS_PER_LOT**	3.0	1.0	7.0	3	5	0.6
**PRECARIOUS_SETTLEMENT_LEVEL**	3.0	2.0	8.0	3	5	0.6
**HDI-INCOME**	4.0	3.0	8.0	1	5	0.2
**VEGETATION_COVERAGE**	4.0	3.0	8.0	1	5	0.2
**LONGITUDE**	5.0	1.0	8.0	2	5	0.4
**POPULATION_DENSITY**	5.0	4.0	8.0	0	5	0.0
**ACCESS_TO_SQUARES**	6.0	1.0	7.0	1	5	0.2

Table S9C. SHAP Feature Stability – Individual Level (Logistic Regression).

Variable	Median position	Best position	Top k folds	N folds	n_folds	Top k frequency
**SEX_M**	1.0	1.0	7.0	4	5	0.8
**CASTRATED_Y**	2.0	2.0	5.0	4	5	0.8
**HABIT_ONLY_IN**	4.0	2.0	6.0	2	5	0.4
**ANIMAL HOARDING_Y**	4.0	4.0	5.0	0	5	0.0
**SUSPECTED HOUSEHOLD PETS_Y**	5.0	1.0	9.0	2	5	0.4
**EXPOSURE_Y**	5.0	3.0	9.0	2	5	0.4
**ULCER DISTRIBUTION_WIDESPREAD**	6.0	6.0	9.0	0	5	0.0
**HABIT_ONLY_OUT**	7.0	7.0	8.0	0	5	0.0

Table S9D. SHAP Feature Stability – Individual Level (Radom Forest).

Variable	Median position	Best position	Top k folds	N folds	n_folds	Top k frequency
**SEX_M**	1.0	1.0	8.0	4	5	0.8
**CASTRATED_Y**	2.0	2.0	3.0	5	5	1.0
**ANIMAL HOARDING_Y**	3.0	1.0	7.0	3	5	0.6
**HABIT_ONLY_IN**	4.0	3.0	7.0	1	5	0.2
**ULCER DISTRIBUTION_WIDESPREAD**	5.0	5.0	6.0	0	5	0.0
**SUSPECTED HOUSEHOLD PETS_Y**	6.0	2.0	8.0	1	5	0.2
**EXPOSURE_Y**	6.0	4.0	8.0	0	5	0.0
**HABIT_ONLY_OUT**	7.0	3.0	9.0	1	5	0.2
**KITTEN_Y**	9.0	6.0	9.0	0	5	0.0

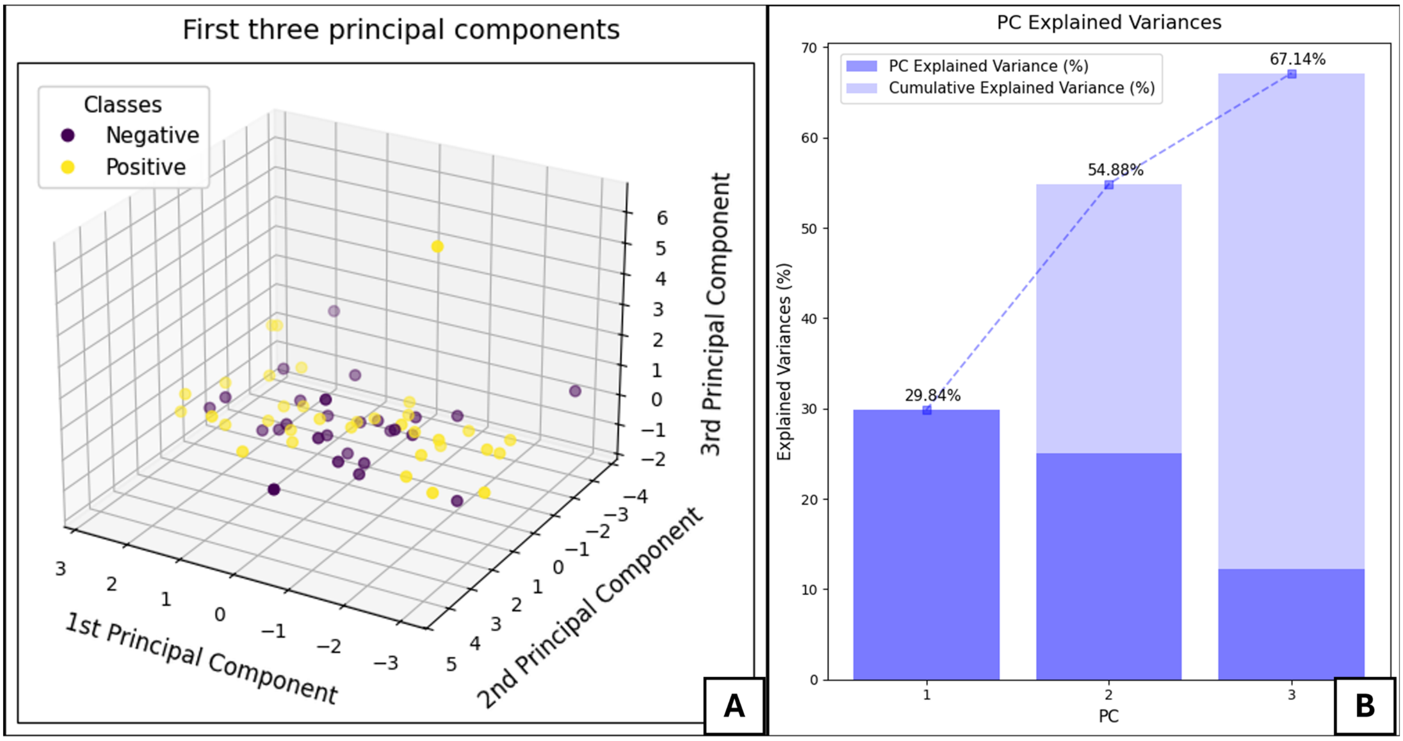

Figure S2. Principal component analysis of neighborhood-level predictors: (A) projection onto the first three principal components, with observations colored by outcome class; (B) individual and cumulative percentages of explained variance.

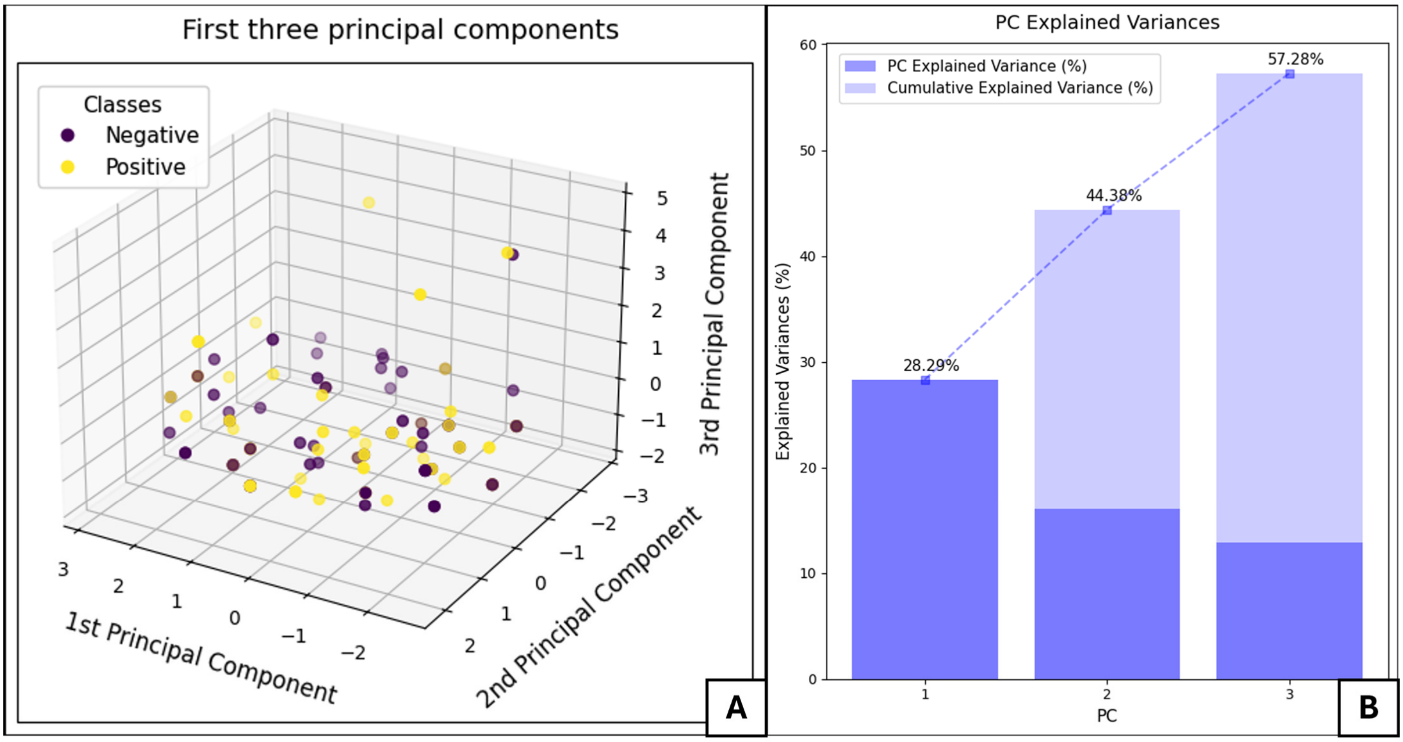

Figure S3. Principal component analysis of individual animal-level predictors: (A) projection onto the first three principal components, with observations colored by outcome class; (B) individual and cumulative percentages of explained variance.

Table S10. Principal Component Analysis (PCA) loadings and proportion of explained variance for the first three principal components (PC1–PC3) across individual-level and neighborhood-level predictor sets.

Analysis Criteria	Component	Variance Explained (%)	Coefficient	Predictor Indicator
Individual, complete-cases	PC1	28.3%	0.212	SEX_M
Individual, complete-cases	PC1	28.3%	-0.295	CASTRATED_Y
Individual, complete-cases	PC1	28.3%	-0.091	KITTEN_Y
Individual, complete-cases	PC1	28.3%	-0.467	HABIT_ONLY_IN
Individual, complete-cases	PC1	28.3%	0.422	HABIT_ONLY_OUT
Individual, complete-cases	PC1	28.3%	0.413	SUSPECTED HOUSEHOLD PETS_Y
Individual, complete-cases	PC1	28.3%	0.489	EXPOSURE_Y
Individual, complete-cases	PC1	28.3%	0,218	ANIMAL HOARDING_Y
Individual, complete-cases	PC1	28.3%	-0.080	ULCER DISTRIBUTION_WIDESPREAD

Individual, complete-cases	PC2	16.1%	0.458	SEX_M
Individual, complete-cases	PC2	16.1%	-0.457	CASTRATED_Y
Individual, complete-cases	PC2	16.1%	-0.121	KITTEN_Y
Individual, complete-cases	PC2	16.1%	-0.164	HABIT_ONLY_IN
Individual, complete-cases	PC2	16.1%	0.085	HABIT_ONLY_OUT
Individual, complete-cases	PC2	16.1%	-0.398	SUSPECTED HOUSEHOLD PETS_Y
Individual, complete-cases	PC2	16.1%	-0.164	EXPOSURE_Y
Individual, complete-cases	PC2	16.1%	-0.331	ANIMAL HOARDING_Y
Individual, complete-cases	PC2	16.1%	0,488	ULCER DISTRIBUTION_WIDESPREAD
Individual, complete-cases	PC3	12.9%	-0.021	SEX_M
Individual, complete-cases	PC3	12.9%	-0.418	CASTRATED_Y
Individual, complete-cases	PC3	12.9%	0.691	KITTEN_Y
Individual, complete-cases	PC3	12.9%	0.052	HABIT_ONLY_IN
Individual, complete-cases	PC3	12.9%	0.270	HABIT_ONLY_OUT
Individual, complete-cases	PC3	12.9%	-0.204	SUSPECTED HOUSEHOLD PETS_Y
Individual, complete-cases	PC3	12.9%	-0.255	EXPOSURE_Y
Individual, complete-cases	PC3	12.9%	0.154	ANIMAL HOARDING_Y
Individual, complete-cases	PC3	12.9%	-0.376	ULCER DISTRIBUTION_WIDESPREAD
Neighborhood, no health units	PC1	29.8%	0.526	LATITUDE
Neighborhood, no health units	PC1	29.8%	-0.164	LONGITUDE
Neighborhood, no health units	PC1	29.8%	0.282	HDI-INCOME

Neighborhood, health units	no	PC1	29.8%	0.313	POPULATION_DENSITY
Neighborhood, health units	no	PC1	29.8%	-0.283	RESIDENTS_PER_LOT
Neighborhood, health units	no	PC1	29.8%	0.487	ACCESS_TO_SQUARES
Neighborhood, health units	no	PC1	29.8%	-0.314	VEGETATION_COVERAGE
Neighborhood, health units	no	PC1	29.8%	-0.321	PRECARIOUS_SETTLEMENT_LEVEL
Neighborhood, health units	no	PC2	25.0%	0.111	LATITUDE
Neighborhood, health units	no	PC2	25.0%	-0.229	LONGITUDE
Neighborhood, health units	no	PC2	25.0%	-0.448	HDI-INCOME
Neighborhood, health units	no	PC2	25.0%	0.525	POPULATION_DENSITY
Neighborhood, health units	no	PC2	25.0%	0.462	RESIDENTS_PER_LOT
Neighborhood, health units	no	PC2	25.0%	0.028	ACCESS_TO_SQUARES
Neighborhood, health units	no	PC2	25.0%	-0.327	VEGETATION_COVERAGE
Neighborhood, health units	no	PC2	25.0%	0.372	PRECARIOUS_SETTLEMENT_LEVEL
Neighborhood, health units	no	PC3	12.3%	0.193	LATITUDE
Neighborhood, health units	no	PC3	12.3%	0.856	LONGITUDE
Neighborhood, health units	no	PC3	12.3%	0.194	HDI-INCOME
Neighborhood, health units	no	PC3	12.3%	0.175	POPULATION_DENSITY
Neighborhood, health units	no	PC3	12.3%	0.012	RESIDENTS_PER_LOT
Neighborhood, health units	no	PC3	12.3%	0.086	ACCESS_TO_SQUARES
Neighborhood, health units	no	PC3	12.3%	-0.050	VEGETATION_COVERAGE
Neighborhood, health units	no	PC3	12.3%	0.390	PRECARIOUS_SETTLEMENT_LEVEL
